# Decoding vascular calcification: mechanistic insights and translational strategies

**DOI:** 10.1007/s00018-026-06086-4

**Published:** 2026-02-10

**Authors:** Hossein Adelnia, Subarna Ray, Hang Thu Ta

**Affiliations:** 1 School of Environment and Science, Griffith University, Nathan, QLD 4111 Australia; 2 Australian Institute for Bioengineering and Nanotechnology, University of Queensland, St Lucia, 4072 Australia

**Keywords:** Vascular calcification, Promoters, Inhibitors, Chelation therapy

## Abstract

Vascular calcification (VC) is a complex, multifactorial process strongly associated with ageing, atherosclerosis, chronic kidney disease, and diabetes. Despite its clinical significance, effective treatment strategies remain elusive due to an incomplete understanding of the underlying mechanisms. In this review, we critically examine the cellular and molecular pathways that drive VC, including the transdifferentiation of vascular smooth muscle cells (VSMCs), the role of extracellular vesicles, and the influence of oxidative stress and inflammation. We also summarise key inducers and endogenous inhibitors of calcification, highlighting therapeutic targets currently under preclinical or clinical investigation. Notably, we evaluate recent advances in pharmacological and biomaterial-based interventions aimed at halting or reversing calcification, with a focus on their translational potential. By integrating mechanistic insights with therapeutic developments, this review offers a comprehensive perspective on VC pathophysiology and treatment, serving as a timely reference for future research and clinical innovation.

**Graphical Abstract:**

TOC Figure

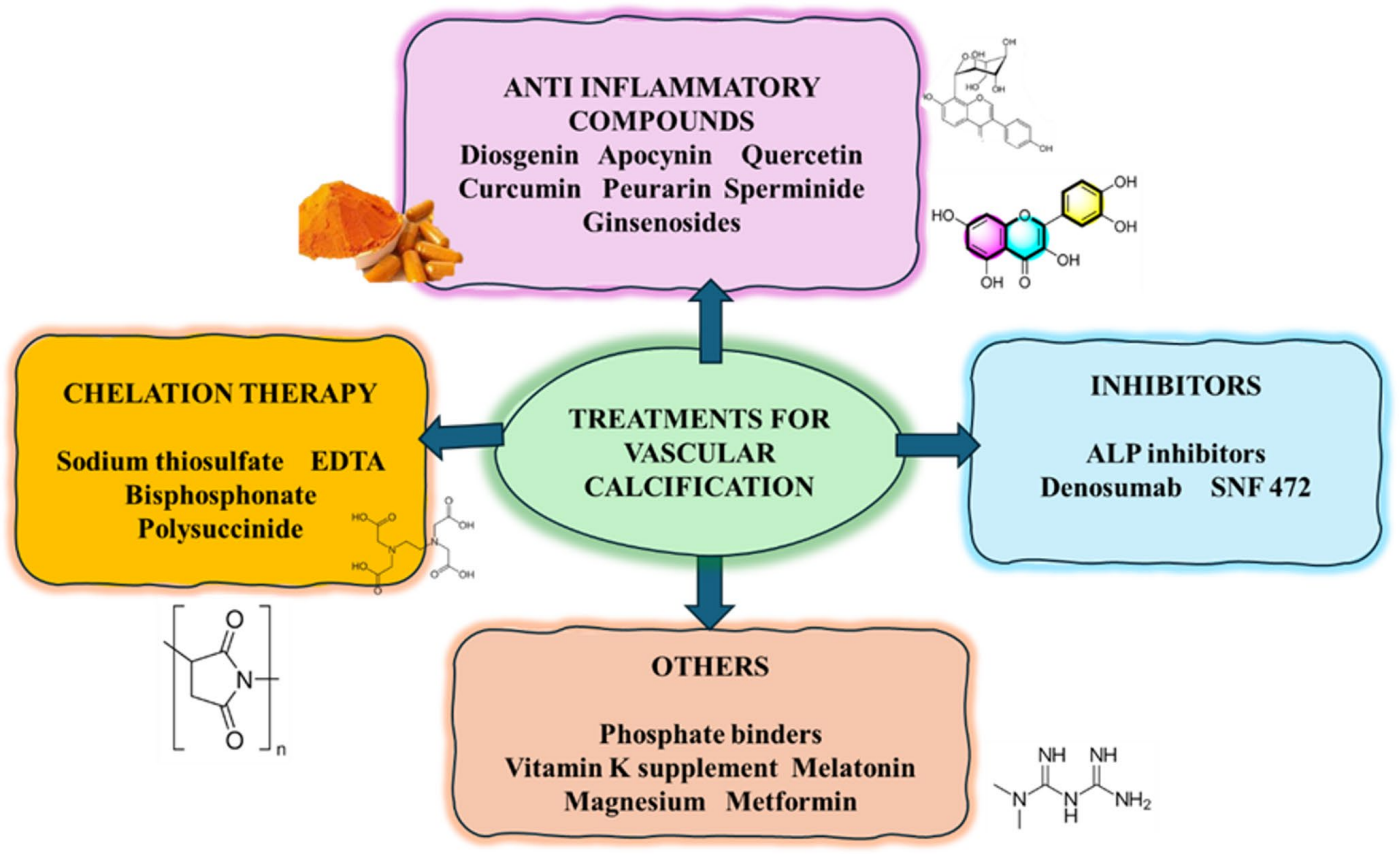

## Introduction

Vascular calcification (VC) refers to the ectopic deposition of calcium in blood vessels in the form of calcium phosphate salts, which are crystallized to form hydroxyapatite (HAp). In addition to the vasculature system, calcification can also occur in other areas such as valves, and even in small vessels in the fatty tissues and skin subcutaneously, which is referred to as calciphylaxis. Early stages of VC can result in high pulse pressure and low coronary perfusion due to high vessel wall stiffness [1]. In advanced stages, it can lead to adverse cardiovascular events such as stenosis, stroke, myocardial infarction (MI), vessel dissection, and rupture during percutaneous coronary interventions [2].

The major cause of morbidity and mortality in patients with end-stage kidney disease and stage 4–5 chronic kidney disease (CKD) who are receiving hemodialysis is calcification [3, 4]. In adult CKD patients older than 45, and younger than 30, end stage kidney disease respectively leads to > 20-times and > 150-times risk of cardiovascular death when compared with an age-matched group [5, 6].

**Fig. 1:**
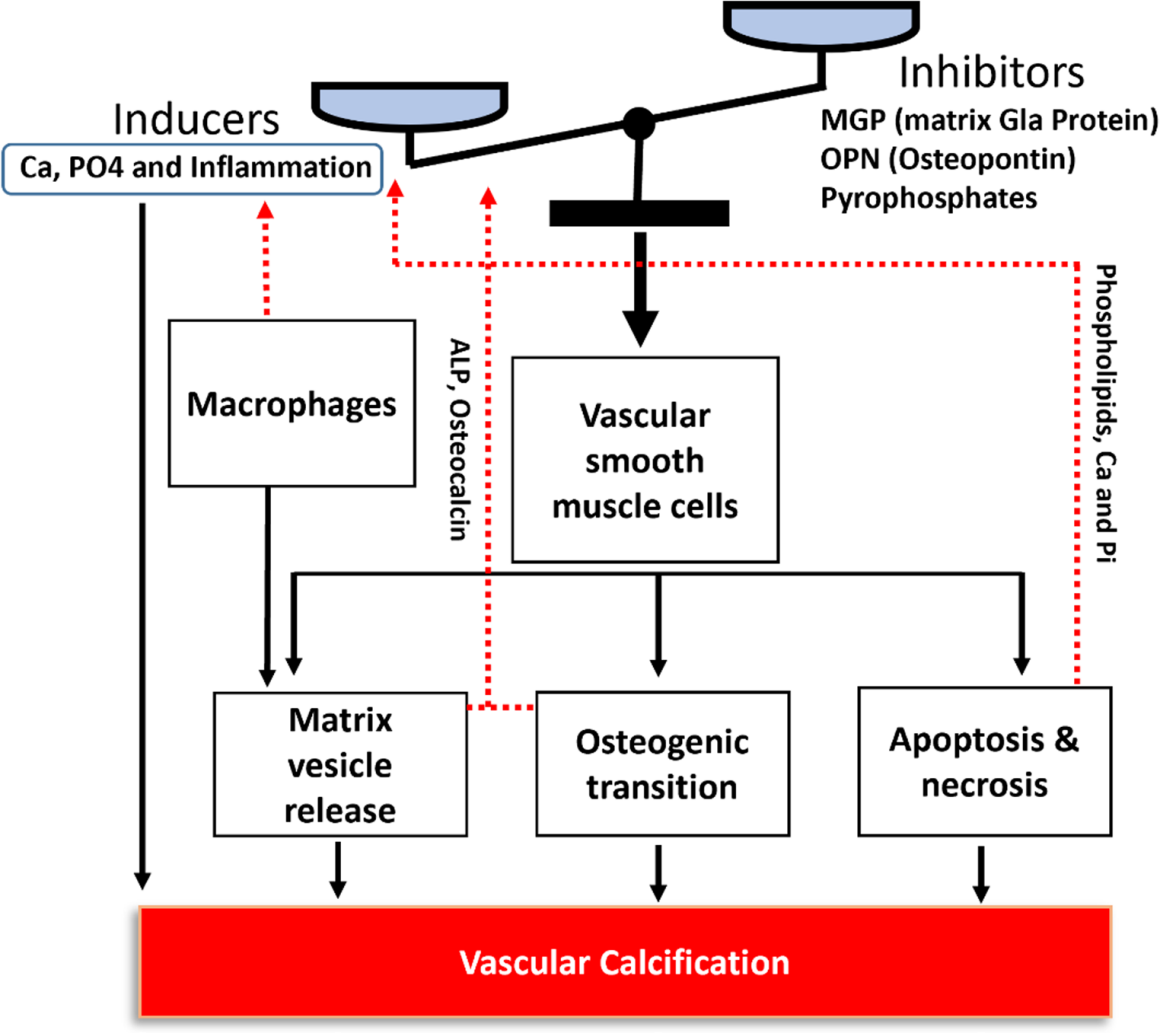
Pathology of VC: The effect of inducers and inhibitors on vascular smooth muscle cells

Calcification was once thought to be a passive result of aging and elastin degradation but is now recognized as an active, regulated process involving vasculature cells and bone-related proteins. High levels of matrix-Gla protein (MGP), osteopontin, osteocalcin, and alkaline phosphatase (ALP), which are expressed in the calcification of skeletal tissues (ossification), have been found within the calcified vessels [7]. The mineral component of both bone and calcific vessels (hydroxyapatite) was also found to be similar in terms of crystallization and structure [8]. Calcification can be regulated by a number of inducers and inhibitors. In general, high serum levels of Ca (hypercalcemia) and P (hyperphosphatemia), loss of inhibitors, high levels of reactive oxygen species (ROS), inflammation, apoptotic bodies, and necrotic debris, as well as trans-differentiation of VSMCs as inducers contribute to calcification. When induction processes dominate inhibition processes, calcification is likely to occur and vice versa (Fig. 1).

Current reviews on the treatment of VC lack detailed discussion of the underlying therapeutic mechanisms and the status of human clinical trials up to 2024. Moreover, there are few studies comparing the drugs with each other; thus, it cannot be determined which intervention is the most promising for treating VC from a clinical perspective [9–11]. This review provides a novel comprehensive analysis of the various forms of VC, categorized by anatomical distribution and etiological origin, alongside their respective molecular mechanisms. Emphasizing recent insights, this review explores the interplay of genetic and molecular contributors to VC pathogenesis in the context of cardiovascular disease (CVD), highlighting their interrelated pathways and identifying emerging therapeutic targets. Central to this discussion is the dysregulation between calcification promoters and inhibitors, a critical factor in modulating disease progression. Furthermore, the review evaluates contemporary treatment strategies, including anti-inflammatory agents, chelation therapy, and inhibitors while detailing their underlying molecular actions. Finally, the translational potential of these therapies is assessed through a synthesis of data derived from in vitro experiments, animal models, and clinical trials. Further research is imperative to elucidate the mechanisms underlying the impact of inducers and therapeutics on VC and develop targeted dietary interventions to prevent or slow this condition. By prioritizing effective therapeutics on VC, we can make significant strides in improving cardiovascular health and reducing the burden of calcification-related diseases (Table 1).

**Table 1:** Treatment strategies forVC– preclinical studies

Therapeutic	Cell line, key in vitro finding and mechanism	In vivo key finding	In vivo administration	Reference
Magnesium	VSMC Magnesium has shown to inhibit VCin human aortic VSMC, increasing transient receptor potential melastatin (TRPM) 7 expression, modulating secretion of VC markers such as osteocalcin and MGP	In animal model of genetically high Mg2+, aorta was protected against calcification and increased expression of the antiosteogenic protein OPNwas observed	Wistar Kyoto (**WKY**) rat (Dosage: 2.0, 2.5, and 3.0 mmol/L for 10 days ) Ex vivo method by isolating aorta	[12, 13]
Magnesium		High phosphate and low magnesium promote calcification	DBA/2 female mice (Dosage: 0.74 g, 1.5 g/100 g body weight/day for 28 days Oral administration	[14]
Magnesium sulfate		Vitamin D and magnesium has shown lower calcium and ALP levels than vitamin D alone	Sprague Dawley rats Intravenous	[15]
Magnesium citrate		Adenine-induced Chronic Renal Failure Rat Model has shown a reduced degree of calcification with reduced calcium content, P levels, ALP activity, and protein levels of RUNX2 and increased protein levels of α-SMA	Male Sprague–Dawley (SD) rats Dosage: 375–750 mg/kg per day for 28 days) Oral administration with high and low magnesium diet	[16]
Curcumin	Rat VSMC Curcumin reduced the calcification by reducing calcium levels, Inhibiting JNK/Bax signalling pathway, decreasing caspase 3 activity, bone-related proteins such as BMP2, Osterix, and Runx2 and ALP levels.			[17]
Curcumin	Tendon stem/progenitor cells. Curcumin reversed the osteogenesis and promoted tendeogenesis with elevated Col1α1, Col3α1, and Col14α1	Curcumin reduced the levels of gene expression of TNF-α, IL-1α, IL-1β, IL-6, MCP-1, MIP-1α and RANTES and increases MCP-1 and MIP-1α expression. Curcumin also shows improved collagen deposition	8-month-old rats Dosage: 3 µg/leg into the right legs subcutaneously every 3 days up to 4 weeks	[18]
Curcumin	Human aortic valve interstitial cells. Increase in ALP and downregulation of RUNX2 by interference with the activation of NF-κB/AKT/ERK pathways.			[19]
Apocynin		Apocynin administration to diabetic rats with VC substantially alleviated arterial calcium deposition. Apocynin inhibited NADPH oxidase activity.	Male Wistar rats Dosage: 2, 5 mg. kg^− 1^. day^− 1^ at 5th week subcutaneous for 18 week period	[20]
Apocyanin	VSMC. Apocynin enhanced expression of a-SMA by 5.3%, and reduced expression of BMP2, Runx2, OPN by 3.37%, 0.61% and 3.07% by suppression of ERK1/2 pathway			[21]
Diosgenin	VSMC. Downregulation of VSMC markers and upregulation of osteochondrogenic transdifferentiation markers such as Pit-1ALP and collagen type 1 by phosphorylation of p38 mitogen-activated protein kinase (p38 MAPK), extracellular signal-regulated kinase (ERK), jun N-terminal kinases (JNK), and protein kinase B (PKB/Akt)	Diosgenin reduced the level of lipid peroxide markers and prevented NO production	Male albino Wistar rats Dosage: 40 mg/kg/day for 5 weeks Oral administration	[22, 23]
Puerarin	VSMC. Prevention of calcium deposition in a dose-dependent manner, inhibition of ROS and NLRP3/Caspase1/IL-1β pathway.	Inhibition of calcium deposition, puerarin significantly inhibits the expression of NLRP3, Caspase1 and IL-1β	Sprague-Dawley (SD) rats Dosage: 400 mg/kg by gavage once a day for 6 weeks Oral administration	[24]
Resveratrol	Rat VSMC. Reduction in mRNA level of FGF-23 and increased the mRNA level of klotho by regulating Sirt-1 and Nrf2 expression in smooth muscle cells			[25]
Quercetin		Decrease in ALP levels, calcium accumulation by modulation of oxidative stress and iNOs/p38MAPK pathway.	Seven-week-old male Wistar rats Dosage: 25 mg/kg/d for 6 weeks Oral administration	[26]
Ginsenoside Rb1	VSMC. Rb1 increased the expression of α-SMA and calponin by activation of peroxisome proliferator-activated receptor-γ (PPAR-γ) and inhibition of Wnt/β-catenin pathway and promotion of osteogenic differentiation	Reduction of calcium deposition as well as ALP activity and calcium concentration in CKD rat arteries	CKD male Wistar rats Dosage: 40 mg/kg/d for 4 weeks Intraperitoneal administration	[27]
Spermidine, a naturally synthesized polyamine	VSMC. Reduction in ALP, BMP-2, and RunX2 by regulation of Sirt-1 and Nrf2	Attenuation of calcium and mineral deposition in aorta, downregulation of BMP2 and Runx2 in aortic arteries	Male Sprague–Dawley (SD) rats Dosage: 3 mM intraperitoneal injection twice a week for 4 weeks	[28]
*Dendrobium officinale* polysaccharide (DOP)	VSMC. DOP reduced the expression of NLRP3, NF-κB, and IL-1β	DOP inhibits calcification in aorta	Adult male SD rats Dosage: 50 mg/kg orally daily for 8 weeks	[29]
TNAP inhibitor, SBI-425		SBI-425 significantly suppressed TNAP activities of aortic tissues, reduced formation of MAC in the abdominal aorta	CKD-mineral and bone disorder (MBD) mouse model Dosage: 30 mg/kg once per day after week 14 of normal diet for 6 weeks Oral administration	[30]
SNF472, hexasodium salt of phytate	VSMC and osteoblast. SNF472 inhibited calcification process by binding to surfaceHAp .			[31]
Denosumab		Denosumab has shown to reduce the calcium content in aorta	Eight-month-old mice Dosage: 10 mg/kg twice weekly for 4 weeks Subcutaneous	[32]
EDTA in PLGA nanoparticle		Removal of calcium from elastin and aorta by chelation without causing vascular damage and change in normal plasma levels of Ca and phosphorus. EDTA binds to metals via four carboxylate and two amine groups	Male Sprague–Dawley rats Dosage: Periadventitial administration of 30 mg of EDTA loaded PLGA nanoparticles pellet for 1 week (injected one time only for 1 week duration)	[33]
EDTA-loaded albumin NPs		Reduction in calcium levels by 3 folds in aorta and reverses calcification. Chelation of EDTA with calcium.	Sprague–Dawley (SD) rats (6–8 weeks old) Dosage: 5 mg of NP/rat/injection Injected through the tail vein two times a week for two weeks	[34]
EDTA-loaded albumin nanoparticles		Reversal of calcification by decreasing calcium content by 40% in aorta. Chelation of EDTA with calcium.	Male Sprague-Dawley rats Dosage: 3 mg/kg body weight twice a week after 4th week for the next 2 weeks. Systemic EDTA injections	[35]
STS		Reduction of calcium content in aorta. Chelation with calcium.	Male Wistar rats Dosage: 0.4 g/kg body weight 3 times a week for 10 weeks Intraperitoneal injection.	[36]
Bisphosphonates		Bisphosphonates can directly inhibit uremic VC independent of bone resorption. Reduction of ALP and calcium levels.	Male Sprague-Dawley rats Dosage: 0.17, 1.7, and 17 mg/kg per day for 28 days Subcutaneous injection	[37]
Bisphosphonates		Reduction of calcification in arteries and heart valves by 4 folds	Male Sprague-Dawley rats Dosage: 0.25 mg P · kg^− 1^ · d^− 1^ for 4 weeks Intravenous	[38]
Polysuccinimide nanoparticle	VSMC. Reduction of ROS levels and calcium by chelation of free calcium ions.	Reduction of calcium in aorta	8weekold male Sprague–Dawley rats Dosage: 50 mg of NPs/kg twice a week for 6 weeks Intravenous injection	[39]
Melatonin	VSMC. Attenuated calcium deposition and reduced ALP levels by activation of AMP-activated protein kinase/mammalian target of rapamycin/Unc-51-like kinase 1 (AMPK/mTOR/ULK1) signaling pathway in VSMC			[40]
Metformin	VSMCs. Metformin treatment reduced VC, expression of RUNX2, BMP2, decreased calcium nodes by activation of AMPK signalling pathway	Increased protein expression of Nuclear factor E2-related factor 2 (Nrf2) and regulated ferroptosis and inhibits expression of Keap1in aortic tissue	Male Sprague-Dawley (SD) rat Dosage: 200 mg/kg/day for 6 weeks Intragastrical administration	[41]

## Classification of VC

**Fig. 2:**
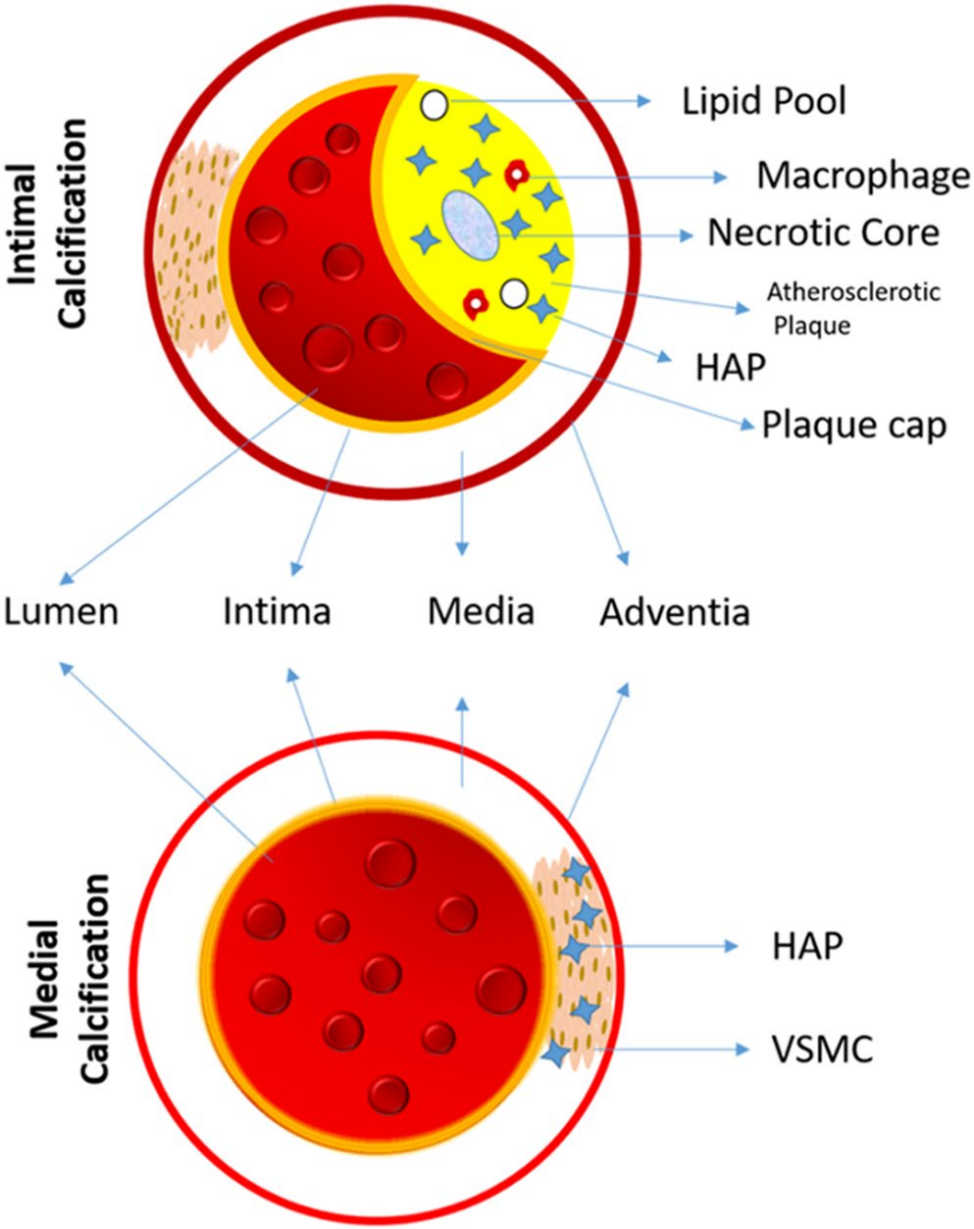
Intimal calcification versus medial calcification; the location of calcium deposition in the former and the latter is intima, medial layer, respectively

Calcifications can be divided into intimal and medial types, depending on their location within a blood vessel. As shown in Fig. 2, the former occurs in areas devoid of smooth muscle cells where endothelial cells and subendothelial connective tissue exist (i.e., intima). The process of calcification of the intima is generally associated with atherosclerosis, in which the intima is highly and chronically inflamed due to endothelial dysfunction. As a consequence, this type of calcification is closely linked to inflammation where mast cells and macrophages play a significant role and considered to be the starting point of the process. Macrophages produce tumour necrosis factor-α (TNF-α), in the presence of oxidized low density lipoproteins (LDL), which in turn induces osteoblastic differentiation of VSMCs and consequently higher level of ALP [42]. Apoptosis of macrophages and VSMCs, as well as the release of matrix vesicle can also intensify calcification [43]. Calcium phosphate in small size particles (< 1 μm) is produced in early stages of intimal calcification.

In contrast, medial calcification (Monckeberg’s sclerosis or arteriosclerosis) occurs along the degraded elastin fibres around smooth muscle cells (Fig. 2). This type of calcification is non-inflammatory, non-occlusive and is generally associated with CKD(CKDs), diabetes, and aging, resulting in stiffening of the vasculature, and hypertension. Vascular smooth muscle cells (VSMCs) play a key role in medical calcification. CKD-induced hypercalcemia and hyperphosphatemia drive VSMC differentiation into osteogenic cells, upregulating bone-forming proteins like ALP and osteocalcin while downregulating contractile proteins, impairing vascular function [44].

## Inducers/Promoters

Several inhibitors and promoters for the VC have been characterized so far [45]. Apart from elevated serum level of calcium and phosphate which can directly and independently lead to precipitation and deposition of calcium phosphate, osteocalcin, bone morphogenetic proteins (BMP-2, 4 and 6), ALP, bone sialoprotein, Runx2, apoptotic bodies, and oxidative stresses, are known to intensify and induce for calcium deposition. Below, some important inducers are discussed.

### High calcium and phosphate levels in the serum

Hyperphosphatemia and hypercalcemia due to mineral imbalance caused by kidney dysfunction are considered the most important inducers of calcification [46]. Free ions of calcium and phosphate are only responsible for complexing. The ionized concentrations of calcium and phosphate in the circulation under healthy conditions are in the range of 1.1–1.3 mM [47], and 0.8–1.45 mM [48], respectively. Under healthy conditions, a large amount (approx. 40% [49]) of total calcium in the serum is complexed, i.e., bound to proteins (primarily albumin [50]), preventing extensive salt precipitation and deposition. The inhibitory role of the proteins synthesized by VSMCs was also demonstrated when in-vitro calcium deposition was compared with cell-free conditions [51].

Even small calcium phosphate particles (calciprotein) have been detected in healthy serum [52]. These tiny particles were suggested to be cleared by the liver sinusoidal endothelial cells [53]. Under pathological conditions, however, the size of these particles greatly increased [54]. The average sizes of calciprotein particles in the serum of CKD patients with and without VC were determined to be 370 nm and 212 nm, respectively. The particle size in healthy volunteers was 168 nm, suggesting a direct correlation between the calciprotein size and calcium deposition [55]. It has been verified in-vitro, where calciprotein particles themselves stimulated calcification of VSMCs through different pathways, further highlighting their importance in the disease etiology [56].

**Fig. 3:**
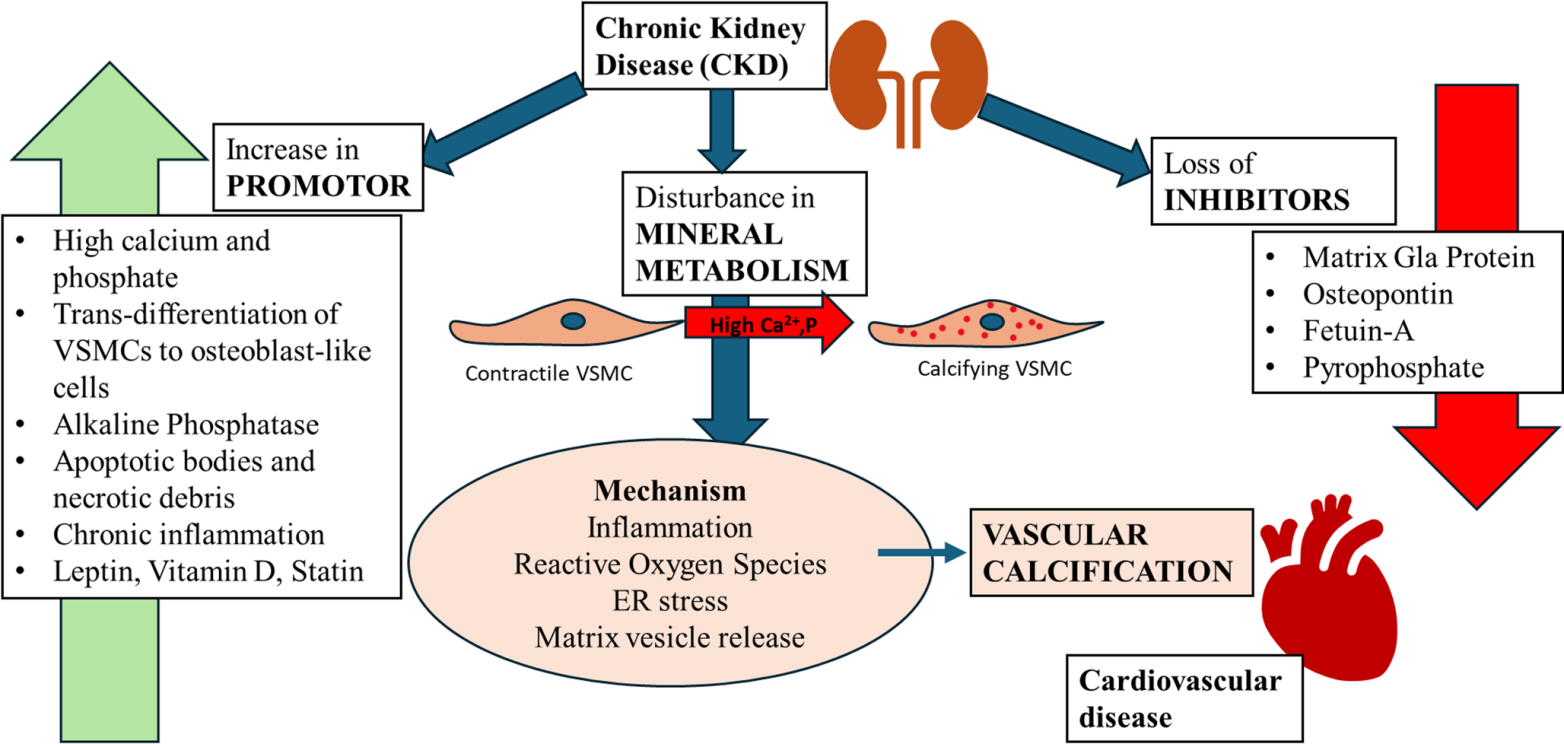
Pathogenesis of VC. Collective role of several inducers such as high calcium, high phosphate, trans differentiation to oseto-/chondroblast-like cells actively accelerate the progression of disease by upregulation of pro-calcification proteins [59]

Passive biological calcification closely mirrors cell-free calcium salt precipitation, with similar influencing factors. Acidic conditions reduce, while alkaline conditions enhance, precipitation—paralleling metabolic acidosis and alkalosis effects [57, 58]. Beyond passive roles, hypercalcemia and hyperphosphatemia actively drive calcification through mechanisms like cell apoptosis and VSMC osteogenic trans-differentiation. (Fig. 3).

### Trans-differentiation of VSMCs to osteoblast-like cells

VSMCs maintain vascular elasticity by contracting and relaxing, regulating lumen diameter and blood pressure. However, in medial calcification, they may transdifferentiate into osteoblast-like cells, losing their contractile function. This osteogenic shift is marked by reduced contractile proteins, increased proliferation and migration, and upregulation of bone-forming proteins (e.g., osteocalcin, ALP, osterix) alongside decreased inhibitors (e.g., osteopontin, MGP, pyrophosphates), promoting HAp nucleation and calcification [60, 61].

Hypercalcemia and hyperphosphatemia are primary drivers of this transition, as shown in vitro, where β-glycerophosphate increases ALP activity, inducing mineral deposition [62]. Interestingly, osteogenic differentiation is reversible under conditions favoring VSMC phenotype restoration [60]. Inflammation and chronic ROS production further accelerate trans-differentiation, with tissue non-specific alkaline phosphatase (TNAP) playing a key role. Notably, atherosclerotic plaques exhibit calcification before osteogenic markers emerge, suggesting inflammation precedes VSMC transition in late-stage atherosclerosis [63].

As mentioned earlier, diabetes is a risk factor for calcification as well. The osteogenic differentiation of VSMCs in primary human aortic VSMCs was promoted by high glucose levels through upregulation of glucocorticoid-inducible kinase 1 (SGK1) [64]. However, treatment of the cells with an SGK1 inhibitor prevented differentiation. Likewise, in another study, similar results were observed in terms of cell differentiation under high glucose conditions which was attributed to IL-1β activation [65]. Recent in-vitro studies on mouse aortic VSMCs (MOVAS) have shown that upon glucose treatment, the ALP level, known as an osteoblast marker, rapidly increased [66]. Apelin-13 attenuated the glucose-induced calcification by suppressing trans-differentiation through inhibition of ROS generation, thereby regulating MAPKs and AKT pathways, and preventing DNA damage. In patients with type 2 diabetes, a direct relationship between plasma hypoxia-inducible factor 1 (HIF-1α) and calcium deposition has been observed as well [67]. Activation of HIF-1α has led to the osteogenic differentiation of pulmonary VSMCs [68]. Overall, one can also conclude that trans-differentiation is the consequence of detrimental impacts of other calcification stimulants and occurs mostly at the latest stages of both CKD and atherosclerosis at which the progression of calcification is highly accelerated [69].

### Alkaline phosphatase (ALP)

ALP, an 86 kDa enzyme primarily expressed by osteoblasts, plays a key role in bone formation and mineralization. Its activity increases at high pH, releasing phosphate for bone growth. ALP hydrolyzes pyrophosphates (PPi), potent calcification inhibitors, into inorganic phosphates, promoting mineralization [70]. Pyrophosphates (PPi), which are potent and natural calcification inhibitors, are hydrolysed by ALP into inorganic phosphates, which are calcification inducers [71]. The inverse relationship between ALP and PPi is well documented. In uremic rat models of calcification, as ALP increased, PPi decreased [71]. In contrast, low ALP level is associated with a high level of PPi [72], and deficient skeletal mineralization [73]. Serum ALP level of patients with calciphylaxis in end-stage kidney failure was found higher than healthy group [74]. Nevertheless, ALP level of plasma may not be an accurate representation of PPi hydrolysis because ALP is present in different tissues at different levels [75].

ALP also dephosphorylates osteopontin (OPN), reducing its anti-calcification properties [76]. A correlation between ALP and OPN has been noted in stage 5 CKD patients, though OPN phosphorylation status remains unexamined [77]. The osteogenic trans-differentiation of VSMCs is marked by ALP upregulation, with long-term b-GP exposure in vitro increasing ALP while downregulating VSMC markers like SM22α and smooth muscle α-actin [78].

### Apoptotic bodies and necrotic debris

In addition to other pathological condition such as chronic inflammation, apoptosis could originate from high calcium and phosphate levels of serum, thereby contributing to calcification process [79, 80]. Apoptosis was described as a process that complements VCrather than being a mutually exclusive pathway by itself [81]. Apoptotic bodies and necrotic debris from macrophage and VSMC particularly increase local concentration of Ca and PO_4_, and initiate nucleation of HAp. Phospholipids from dead cells also function as nucleation agent for HAp crystallization [82, 83]. Matrix vesicles, which are also rich in Ca and PO_4_ are released from apoptotic bodies [84].

In chondrocytes, apoptotic bodies contained a significant amount of ALP and nucleoside triphosphate pyrophosphohydrolase (NTP) as important calcification promoters, which are typically manifested in osteoarthritis [85]. Acceleration of atherosclerotic plaque growth was observed with chronic apoptosis of VSMC in ApoE deficient mice [86]. In high phosphate-induced calcification of VSMCs, iron-based phosphate binders were found to reduce calcification by preventing apoptosis [87].

### Chronic inflammation

**Fig. 4:**
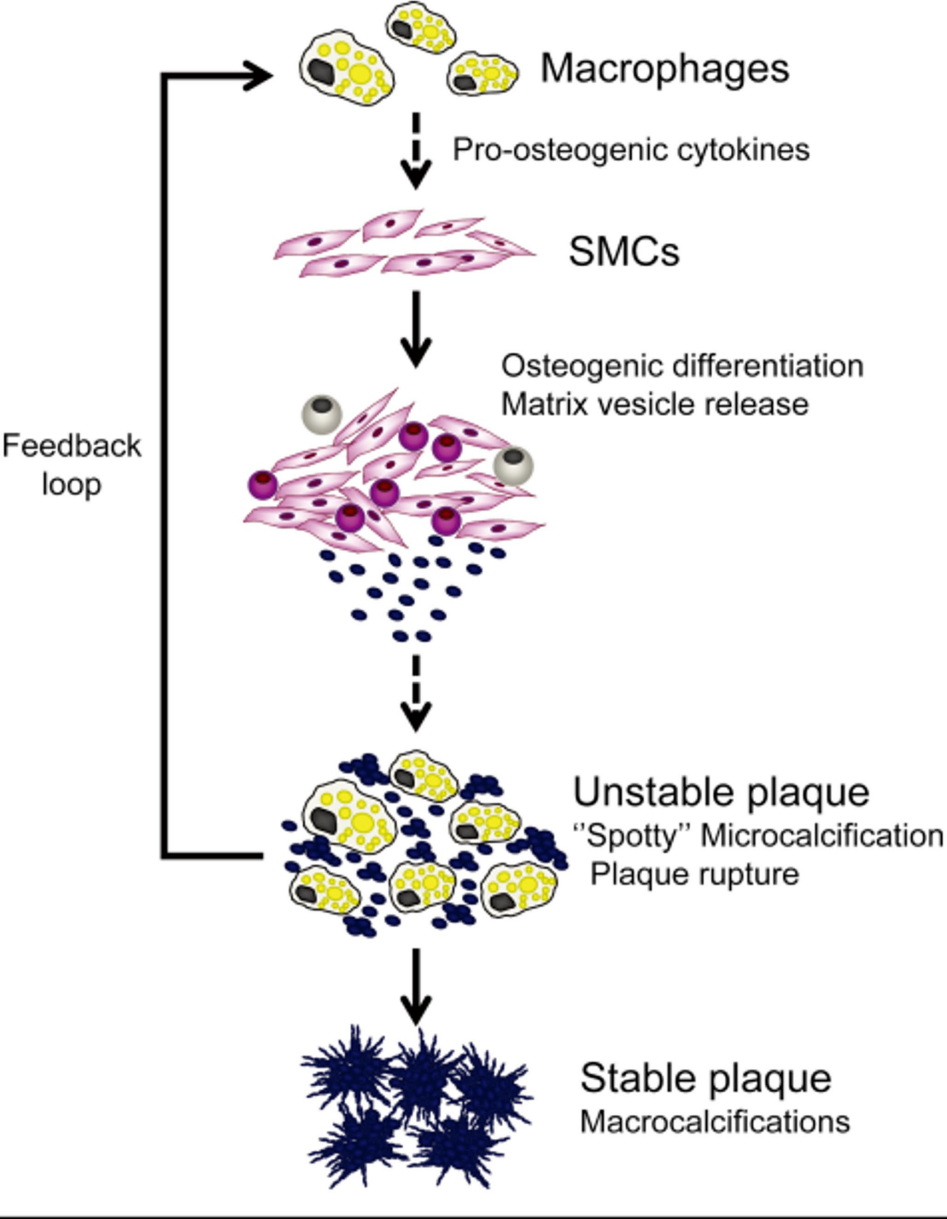
Effect of chronic inflammation and pro-osteogenic cytokines on the initiation and progression ofVC. Inflammation stimulates release of matrix vesicles which ae rich in tiny calcium phosphate particles, leading to micro-calcification. This in turn leads to apoptosis, leaving phospholipid debris and calcium among other promoters, further intensifying inflammation and accelerating the disease [88]

Several studies have demonstrated the relationship between chronic inflammation and osteogenic trans-differentiation of VSMCs. Inflammation stimulates release of matrix vesicles which are rich in tiny calcium phosphate particles, leading to micro-calcification. This in turn leads to apoptosis, leaving phospholipid debris and calcium among other promoters, further intensifying inflammation and accelerating the disease (Fig. 4) [88]. Interleukin-6 (IL-6) upregulation in particular has been found as an important indicator for chronic inflammation in VC [89]. Plasma levels of IL-6, TNF-α, and C-reactive proteins (CRP), as key inflammatory biomarkers, were elevated in CKD patients [90]. Among different biomarkers, IL-6 was found to be a better predictor of mortality rate in CKD patients under haemodialysis. Increased IL-6 was directly correlated with intimal and medial calcification in these patients [91]. Noteworthy is the fact that these cytokines could also contribute to downregulation of fetuin-A, an potent calcium-binding protein in the serum [92]. Furthermore, TNF-α led to a downregulation of Klotho, both in the kidney and vasculature [93]. The downregulation of both fetuin-A and Klotho can be indicative ofVC.

Human coronary arteries from atheroma type III onwards exhibited a significant expression of CD68, a highly expressed protein in chronic inflammation. In the early stages of atherosclerosis, small calcium depositions in the lesions were detected, which grew in size with plaque progression [69]. Increasing CD68 levels were correlated with microcalcification in the lesion. Furthermore, in-vitro, pro-inflammatory role of the calcium deposits was also indicated in the same study. Inflammation leads to calcification and calcification worsens inflammation further. Considering all the above, inflammation in general, and its biomarkers such as IL-6 in particular could be targeted for the treatment VC [94, 95].

### Leptin

Leptin is an enzyme found mostly in the small intestine and blood circulation, whose main function is regulation of fat storage and energy balance [96]. The leptin level is elevated due to kidney dysfunction as it cannot be filtered and cleared from the serum efficiently [97, 98]. Receptors of leptin were characterized in mouse blood vessels [98]. Leptin increased ALP activity 5–10 times in calcifying vascular cells [98]. Long-term treatment of the cells with leptin led to their mineralization [97, 98]. Treatment of endothelial cells with leptin resulted in the generation of oxidative stress, thereby upregulating production of such as BMP-2 [99, 100]. Similarly, in VSMC, leptin increased bone markers such as BMP-2 and Runx2 [90]. Osteoclast generation and thus bone resorption were inhibited by leptin [101]. Overall, in-vitro studies suggest osteoblastic differentiation as a result of leptin, leading to soft tissue calcification.

In different animal models and human trials, the role of leptin has also been indicated. In ApoE-knockout mice models of atherosclerosis, leptin treatment led to 8- and 2.5-fold increase in lesion and valvular calcification as compared to control mice group, respectively [102]. ALP activity was also increased by about 5 times in leptin-treated mice, accompanied with osteogenic differentiation of VSMCs. In a pilot study in patients with type 2 diabetes mellitus who had median calcification score of 100 to 300, leptin level in plasma was found significantly high [90]. In a study involving 548 men aged 50–85 years, high leptin level were associated with severe abdominal aortic calcification [103].

### Vitamin D

**Fig. 5:**
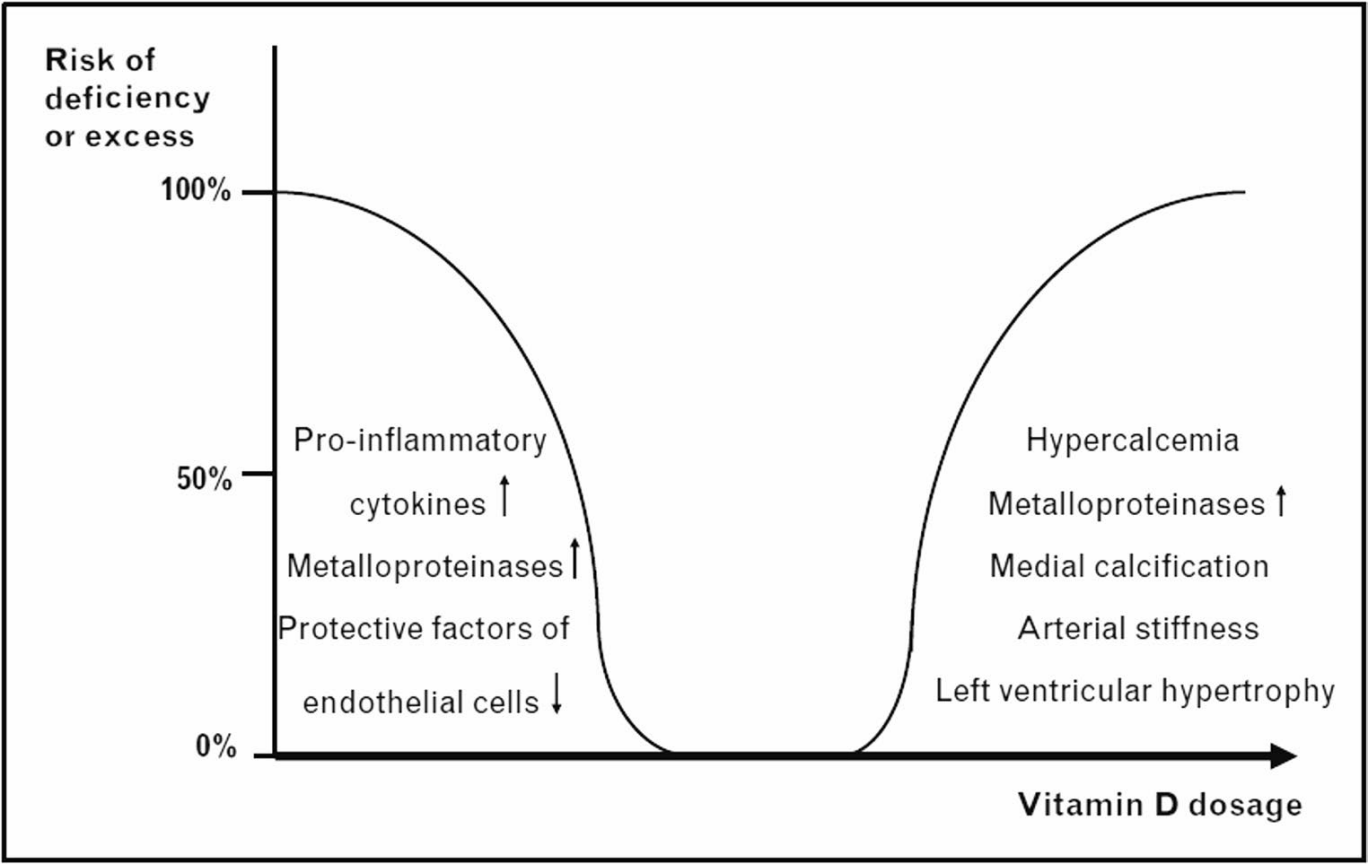
The effect of vitamin D dosage on VC [109]

Among other functions, vitamin D is essential for the absorption of calcium and other minerals. Both low and high vitamin D levels can contribute toVC. (Fig. 5). Its low level can induce chronic inflammation in the vasculature through increasing 1-α hydroxylase activity in endothelial cells, thereby over-proliferating macrophages, increasing cytokine level, overexpressing metalloproteinase and in turn adversely affecting VSMCs [104, 105]. Conversely, a high level of vitamin D increases serum calcium levels significantly and promotes salt deposition [106]. Diets with high level of vitamin D are utilized to induce VCin animal models [107, 108].

### Statins

Statins, inhibitors of HMG-CoA reductase, are regarded as the gold standard for the treatment of atherosclerosis and hypercholesteremia. Statins reduce the cholesterol produced by the liver through blocking HMG-CoA enzyme. Nevertheless, there have been a number of in-vitro, in-vivo, and human trials studies showing that statins may serve as a double-edged sword, as they intensify calcification. Trion et al. demonstrated that atorvastatin (2–50 µM) increases calcification of VSMCs in a dose-dependent manner [110]. Atorvastatin at high concentration (50 µM) reduced cell proliferation, and increased apoptosis, which could be the reasons for calcification as discussed above. In a study on 197 patients with type 2 diabetes, frequent statin administration significantly increased the calcification of the abdominal aortic artery [111]. Despite lowering the risk of cardiovascular events, statin therapy in other larger clinical studies has also been associated with increased calcification [111–114].

### Matrix metalloproteinase (MMP)

Matrix metalloproteinases (MMPs) are a family of zinc-dependent endopeptidases that play a crucial role in extracellular matrix (ECM) remodelling. In VC, MMPs are increasingly recognized as key regulators due to their involvement in VSMC phenotypic changes, ECM degradation, and promotion of mineral deposition. MMP2, MMP9, MMP13, MMP3 have shown to degrade type 1 and 3 collagen, induce ECM breakdown and inflammation, enhances VSMC osteogenic differentiation [115, 116]. MMP activity promotes phenotypic switching of VSMCs from a contractile to an osteogenic phenotype, characterized by upregulation of Runx2, ALP, and OPN [117]. A recent study by Xie et al., revealed that deletion of smooth muscle cell-specific MMP 3 significantly reduced medial calcification and osteogenic transformation [118]. Tissue inhibitors metalloproteinases (TIMPs) such as TIMP-1, -2, -3, and − 4 play major role in MMP regulation and suppress the expression of various MMPs and orchestrate ECM remodelling [119].

### Others

Endoplasmic reticulum (ER) stress promotes calcification through various mechanisms involving VSMCs, and immune cells, and a sudden influx of unfolded proteins leading to an increase in the unfolded protein response (UPR) [120]. Primarily, three key ER stress sensors - IRE1 (inositol-requiring enzyme 1), ATF6 (activating transcription factor 6), and PERK (PKR-like ER kinase) - mediate the UPR by detecting the accumulation of misfolded proteins and activating distinct signalling pathways within the UPR. Activation of UPR leads to a reduction in the entry of newly synthesized proteins into ER and induces the expression of genes encoding molecular chaperones - such as Grp78 (glucose-regulated protein 78), C/EBP homologous protein (CHOP), and c-Jun N-terminal kinase (JNK) - and regulate calcification [121, 122].

Hypoxia-inducible factors (HIFs) facilitates the phenotypic switch of VSMCs toward an osteoblast-like lineage by enhancing the expression of osteogenic regulators, including Runx2, inducing inflammatory factors such as IL-6 and TNF-α thereby contributing to the progression of VC [123]. Other studies have also shown that HIF-1 stimulates bone cell osteogenic differentiation in VSMCs, targets GLUT-1, VEGFA inducing the expression of RUNX2, SOX9 (Sry-related HMG box-9), OCN and ALP in CKD conditions [124–126]. Activation of HIF-1 by Daprodustat, a prolyl hydroxylase inhibitor, has also shown an accelerating medial calcification in CKD patients with hyperphosphatemia [127].

## Inhibitors

Even in a healthy body, as noted, the total level of calcium and phosphate can be high enough for the initiation of its corresponding salt or crystal. However, due to the presence of several inhibitors such as MGP, osteopontin (OPN), fetuin-A and pyrophosphate (PPi), precipitation of calcium phosphate does not occur. These inhibitors basically bind to calcium, forming a complex with them, thereby lowering calcium’s ability to be involved in precipitation and crystallization. From the viewpoint of chemistry, any calcium complexing agent -also referred to as chelator- should have an ionized acidic moiety with negative charges (such as phosphoric and carboxylic acid groups), capable of establishing electrostatic interaction with free calcium cations, lowering its physiologically active (un-complexed) concentration [39, 128, 129].

### Matrix Gla protein (MGP)

**Fig. 6:**
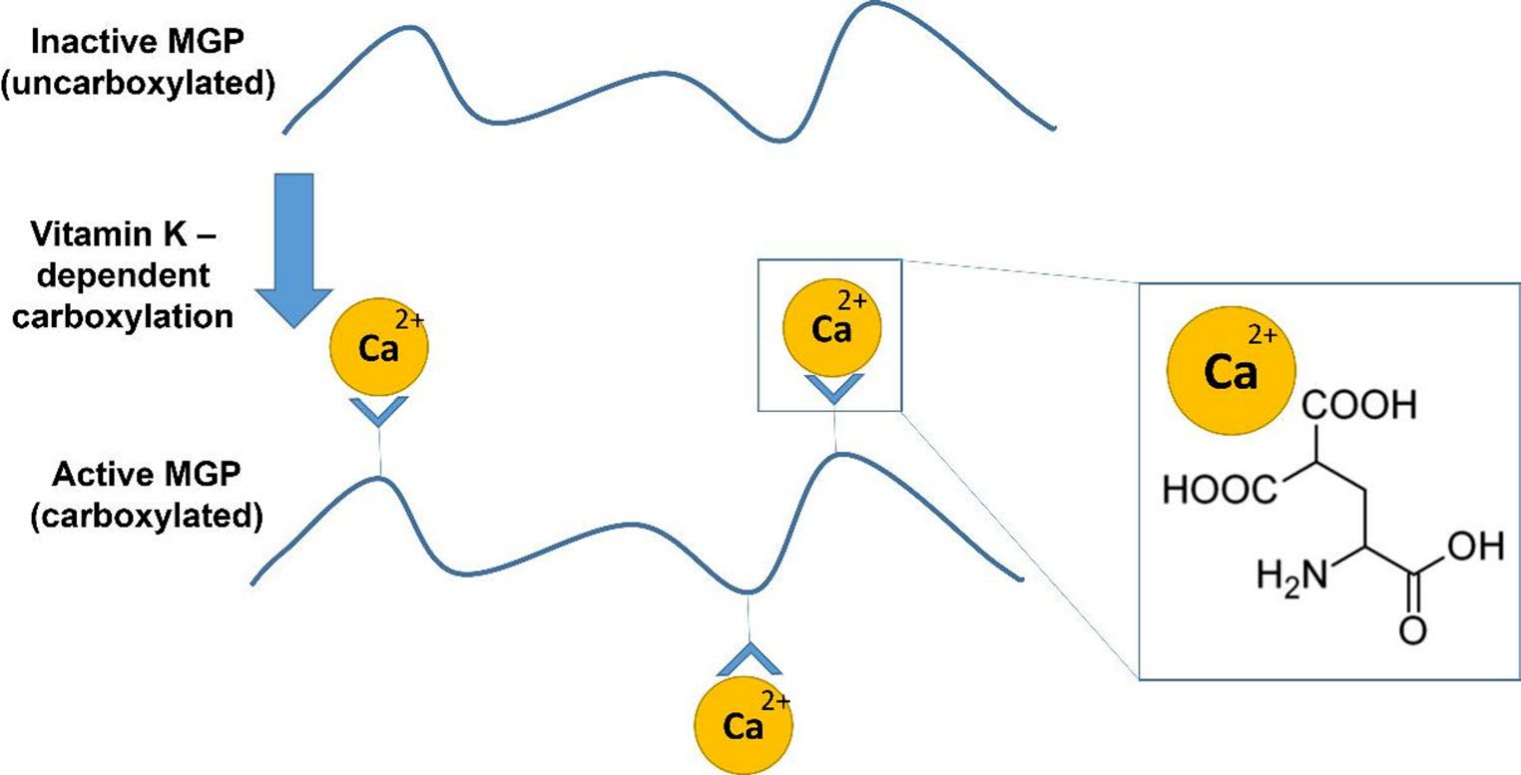
Activation of MGP by vitamin K leads to its carboxylation by introduction of γ-glutamic acid

The role of MGP in CVDin general and VC in particular has been well-understood [130–135]. MGP is a vitamin-K dependent protein synthesized mainly by VSMCs and accumulates in extracellular vesicles [136]. MGP carboxylation, called activation, is largely dependent on vitamin K and thus its deficiency leads to uncarboxylated MGP as shown in Fig. 6. A high affinity of glutamic acid for calcium and metal ions causes activated MGP to bind strongly to calcium ions. Activated MGP also inhibits the synthesis of, BMP-2, a pro-calcifying protein [137]. While hyperphosphatemia does not significantly affect MGP levels in vitro, hypercalcemia downregulates MGP after 48 h [138].

MGP-deficient mice develop severe aortic calcification, leading to fatal hemorrhage within two months. In end-stage kidney diseasepatients, elevated dephosphorylated uncarboxylated MGP correlates with high calcification scores, making it a potential biomarker [130]. (Fig. 6)

### Osteopontin (OPN)

OPN is a phosphoprotein with molecular weight of 33 KDa and has a highly negative charge density due mainly to the presence of large aspartic acid (approx. 15–20%), and glutamic acid resides. OPN is synthesized by a variety of cells including fibroblasts [139], VSMCs [140] and macrophages [141]. In bone remodelling, OPN contributes to activation of osteoclast for initiation of bone resorption (via the α_v_β_3_ integrin) [142]. In urine, OPN, referred to as uropontin (uOPN), is found in large quantities serving as an inhibitor of calcium oxalate monohydrate which is the main constituent of kidney stone [143–145].

InVC, the inhibitory role of OPN was demonstrated and attributed to the prevention of HAp nucleation and growth [146]. OPN also promoted regression of VCin vivo [144]. In comparison to mice lacking MGP alone (MGP^−/−^), mice lacking both MGP and OPN (MGP^−/−^, OPN^−/−^) exhibited much more extensive calcium deposition in the vasculature [147]. Further evidence for the OPN role was obtained in a later research demonstrating that OPN^−/−^ mice fed with high phosphate develops extensive calcification compared to the control mice [145]. Noteworthy is also the fact that bone development in OPN^−/−^ mice occurs stronger as a higher mineral content with larger crystal size were detected [148].

Nevertheless, OPN plays a crucial role in chronic inflammation and is thought to be a biomarker for cancer [149, 150]. While acute expression of OPN has protective role in the vasculature against calcification as explained, chronic OPN overexpression is regarded as a predictor for CVDand its related events, and mortality [151, 152]. Several isoforms of OPN have been characterized in recent years, each of which has its own biological function [153].

OPN synthesized from different cells, under different biological conditions may differ in terms of phosphorylation [154], glycosylation [155], and transglutamination [156]. For example, osteoblast and fibroblast were respectively added 21, and 4 phosphate groups into the OPN structure [154]. Giachelli et al. [76] indicated the considerable role of phosphorylation in inhibition of calcification of VSMCs in-vitro. Nevertheless, the role of aspartic acid residue in OPN should not be neglected or underestimated. Aspartic acid is known as the strongest amino acid for complexation with calcium and HAp [157]. Similarly, poly(aspartic acid), homopolymer of aspartic acid, has shown to inhibit and even dissolve of calcium oxalate and HAp [158–160].

### Fetuin-A

Fetuin-A, as a glycoprotein synthesized in the liver, and abundant in serum, is considered the main carrier of free fatty acids in the bloodstream [161, 162]. Fetuin-A can bind calcium and Calcium phosphate precursor, inhibiting nucleation and growth of HAp [163]. If bound to calcium particles (calciproteins), Fetuin-A can remove these highly insoluble particles from the circulation. The function of Fetuin-A has been attributed to its high negative charge density arising from glutamic acid and aspartic acid residues [164]. The former and the latter are respectively responsible for inhibition activity of MGP and OPN. Fetuin-A is loaded into matrix vesicles, where local calcium level is high, inhibiting the calcium phosphate precipitation, and improving VSMC survival [165]. Fetuin-A-deficient mice fed with a mineral- and vitamin D-rich diet developed extensive calcification [166]. Rats with calcification had detectable fetuin-mineral complexes in their serum, while those treated with ibandronate without calcification had no detectable complex [167]. Low level of fetuin-A has been well documented in dialysis patients and has been associated with high rate of cardiovascular-related mortality [168]. The Fetuin-deficient serum was reconstructed by spiking different amount of Fetuin-A, showing reduced precipitation of calcium in-vitro [166]. Overall, these findings further suggest calcium binding ability of fetuin-A, its affinity toward HAp and its role in inhibition of calcification.

### Pyrophosphate (PPi)

Pyrophosphates, also referred to as diphosphates, are molecules that have a P-O-P bond. They are capable of establishing complexes with metal ions such as calcium and are natural inhibitors for ectopic calcification and HAp crystallization [169]. PPi is found in plasma and its role in the prevention of VC has been demonstrated in-vitro [170], in-vivo [171, 172], and in human trials [173].

 Ex-vivo studies on the cultured rat aortas ring, showed endogenous production of PPi. Addition of ALP to the culture medium led to deactivation of the PPi [174]. It is generally accepted that ALP level is inversely related to PPi level. Through VSMCs trans-differentiation, ALP is upregulated, leading to the hydrolysis of PPi into inorganic phosphate. In other words, one can deduce that ALP converts a strong inhibitor (PPi) into an inducer (inorganic free phosphate).

In another study, the aorta of mice lacking ectonucleotide pyrophosphate phosphodiesterase (ENPP1), an enzyme that produces PPi, developed extensive calcification. Additionally, there is evidence that the loss of ENPP1 function in infantile genetic diseases can also lead to VC in humans [175]. ENPP1-deficient children usually die within six months after birth due to heart failure. PPi level of the patients’ serum with CKD after hemodialysis was found to be lower than that of healthy group because it is removed by the dialysis process [75].

These studies collectively demonstrate the inhibitory role of pyrophposphates based on therapeutic strategies could be designed. However, PPi is typically degraded in the stomach, necessitating their parental infusion. PPi’s half-time in the circulation is shorter than 30 min, which limits its clinical use. The synthetic analogues of PPi, i.e., bisphosphonates could address these challenges which will be discussed below. Overall, one can conclude that maintaining high PPi level in the circulation by targeting its metabolism (e.g., use of ALP inhibitors), rather than its administration could have promising prospects.

## Treatments

In contrast to lipid-laden plaques, calcified plaques are known to be irreversible. One of the most vulnerable and at-risk populations to VC is CKD patients whose kidneys are not able to remove excess minerals. Other at-risk populations include those with atherosclerosis, post-menopausal women, as well as elderly people. Therefore, managing the underlying conditions such as CKDs, hypertension, and diabetes is the first main treatment prescribed. While they could be beneficial in the early stages of calcification, at the latest stages, when major vascular blockage happens, invasive interventions such as percutaneous angioplasty, and metal stenting are adopted. Although such interventions are successful in atherosclerotic plaques, they could be associated with serious complications in the calcified plaques, due to vessel rigidity, posing the vessel dissection risk, and severe haemorrhage A novel method has recently been adopted that uses the principle applied in the fragmentation of kidney stones with strong shockwaves, referred to as lithotripsy, where high-pressure shocks (50 atm, for short pulses) disintegrate large calcium deposits, facilitating further angioplasty and deploying drug-coated balloons [176]. Despite the progress in invasive methods, there is still no clinically approved non-invasive treatment for VC and thus much attention is paid to the underlying causes as stated above to decelerate the disease progression. Understanding the disease, and the mechanism of action of inducers and inhibitors however have led to the development of several therapies examined in-vitro, in-vivo, and in clinical trials. Some of these therapies include but are not limited to phosphate binders, vitamin K supplementation, sodium thiosulfate, etc. which will be discussed as follows. Of note, however, is that none of these therapies have yet been approved or established although prescribed by some practitioners.

Undoubtedly, there is close interface between bone remodelling and VC which should be considered in the design and development of drugs. Off-target action of the drug could potentially lead to bone resorption and osteoporosis, in addition to being ineffective in the prevention of VC. This highlights the utmost importance of drug targeting to the vasculature, and other at-risk organs. Nevertheless, scrutinizing the literature revealed only a few case studies where the active compound was loaded into a nanocarrier and conjugated with elastin antibody. The antibody improved targeting and thus the treatment efficacy which will be further discussed below.

### Phosphate binders

**Fig. 7:**
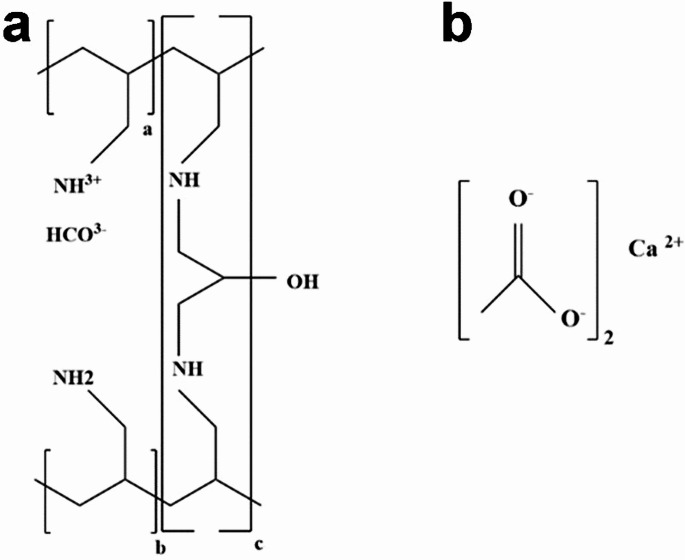
Chemical structure of (**a**) sevelamer (a cross-linked poly(allyl amine) in which carbonate ion is introduced for neutralization, and (**b**) calcium acetate as two phosphate binders used for lowering serum level of phosphate

Phosphate binders are a class of orally-administered drugs that reduce dietary phosphate absorption by forming an insoluble complex with it in the gastrointestinal tract, thereby lowering the serum phosphate level [177, 178]. Their effect has been studied in several animal and human studies [179]. Based on the chemical structure, phosphate binders can be divided into two: (i) calcium-containing and (ii) calcium-free [180]. The most notable examples for the former and the latter are calcium acetate and Sevelamer carbonate (Renagel), respectively (Fig. 7).

Calcium-containing binders, while effective in lowering Pi level, have been shown to even intensify calcification in human trials [181]. However, calcium-free binders effectively inhibited or delayed calcium deposition [182, 183]. A recent human trial compared calcium acetate with Sevelamer, further verifying the superiority of the latter for reducing coronary artery calcification scores, as well as prevalence of hypercalcemia [184]. Sevelamer was also significantly more effective than calcium-containing binders in improving survival rate in haemodialysis patients [185].

From the polymer chemistry viewpoint, Sevelamer is poly(allylamine), a cationic polymer, cross-linked with epichlorohydrin. The amine groups of the polymer are fully, and partially protonated under acidic media of the stomach, and neutral conditions of the small intestine, respectively. The protonated groups form strong electrostatic complexes with free dietary phosphate anions which are then excreted from the body without absorption. Sevelamer should be administered orally 3 times a day for each meal (0.8–1.6 gr depending on the diet). Its expensiveness as well as gastrointestinal side effects restrict its clinical use. A randomized controlled trial on CKD patients to study the effect of sevelamer was performed. The study showed that sevelamer reduced the reduction in systemic, vascular, and bone-related inflammatory markers such as IL-6, IL-8, IL-10, CRP, TNFα, and IFN-γ from treatment with sevelamer in CKD patients. Sevelamer treatment also resulted in a significant decrease in levels of FGF-23, calcidiol, and calcitriol, whereas FGF-23 and calcitriol remained unchanged [186]. Also, as suggested, a well-designed randomized clinical trial is required to confirm whether phosphate binders compared to placebo do indeed attenuate the clinical end point in CKD patients [187].

### Magnesium

**Fig. 8:**
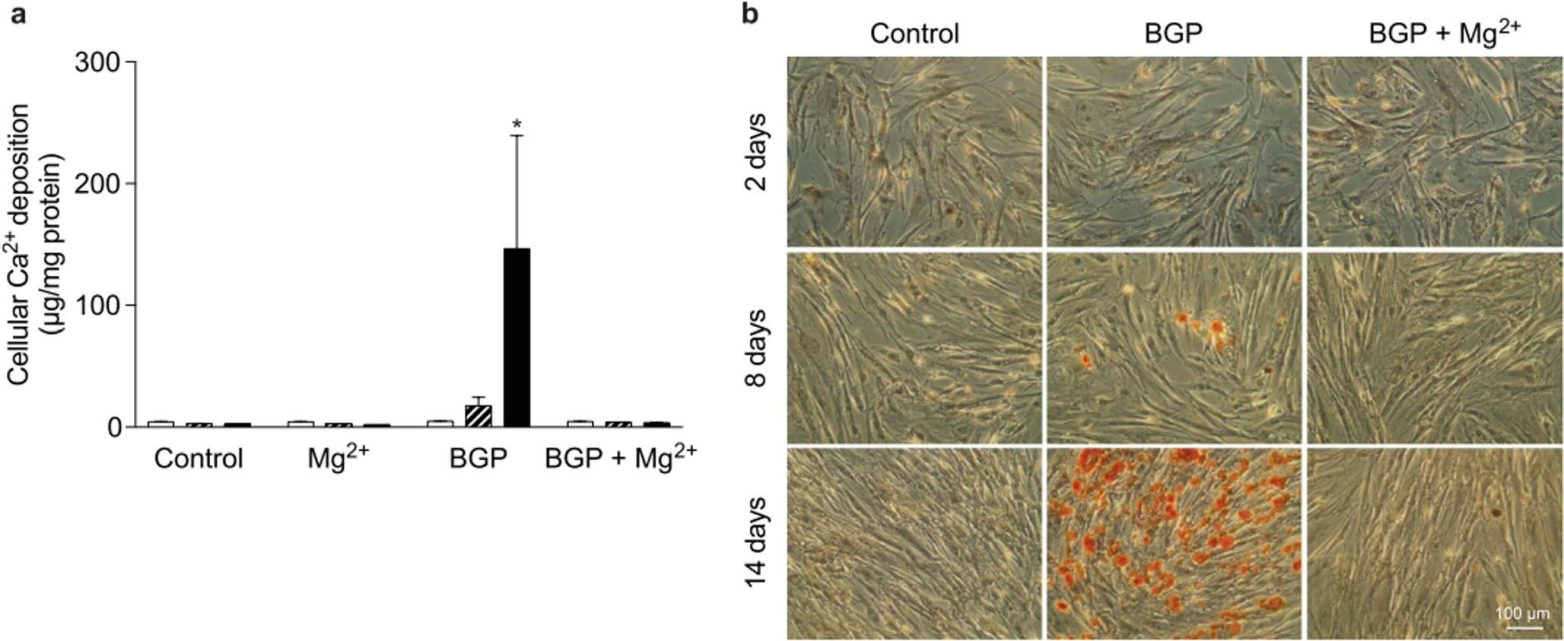
Mg prevents HAp crystallization in the presence of high Pi concentrations. Quantitative and qualitative evaluation of calcification induced by β-glycerophosphate (BGP) at 2 (white bars), 8 (striped bars) and 14 (black bars) days after BGP supplementation (**a**) and Alizarin Red staining of BVSMCs (**b**) [191]

Recently, there have been a great deal of attention to magnesium (Mg^2+^), as many reports have shown its link to cardiovascular mortality in patients with ESRF [188]. Ex-vivo, the synthesis of HAp is inhibited by the presence of Mg ions in the solution [189, 190]. In-vitro, magnesium prevented VSMC calcification, reduced BMP-2 activity and downregulated osteocalcin expression, whereas OPN and MGP were upregulated [12]. VSMCs incubated with Mg^2+^ (2 mM) and Pi (3 mM) exhibited much better cell viability and lower level of calcification. The preventive effect of Mg^2+^ was observed in live cells, but in fixed cells extensive calcification was detected [13]. βGP-induced calcification of VSMC was prevented by 2 mM of magnesium, and explained by a simple passive process; extracellular inhibition of apatite crystallization as free magnesium cations binds phosphate anions, disturbing calcium phosphate salt formation (Fig. 8) [191]. Transient receptor potential melastatin 7 (TRPM7) is regarded as the main magnesium channel in VSMCs [192]. Blocking such channels by 2-aminoethyl diphenylborinate (2-APB) did not prevent the anti-calcification role of magnesium, suggesting its intracellular-independent activity.

*In vivo*, high phosphate and low Mg^2+^ diet in mice model of DBA/2 promoted extensive calcification in heart, kidney, and other organs [14]. Treatment of uremia rat induced by vitamin D and nicotine with magnesium sulfate led to a lower ALP activity and lower calcium content in soft tissues [15]. In adenine-fed rats, magnesium citrate also prevented calcification [16]. A 5-year randomized clinical trial study commenced on 2017 will soon reveal the effect of oral Mg supplementation on coronary artery calcification [193]. The same group also showed that by increasing Mg level of the dialysate, the calcification propensity in hemodialysis patients is decreased [194].

### Vitamin K supplementation

Vitamin K is a well-known enzyme cofactor involved in coagulation as well as other signalling pathways and is mainly found in green vegetables such as spinach. Direct correlation between vitamin K uptake and carboxylation of MGP has been well documented. As discussed, activated MGP is a potent inhibitor for calcification due to the presence of γ-glutamic acid residues. Vitamin K antagonists such as warfarin strongly prevent MGP carboxylation and lead to calcification [195, 196]. MGP activation is carried out in VSMCs and the role of warfarin on calcium deposition in VSMCs was corroborated in-vitro [51]. In ApoE^−/−^ mice, warfarin resulted in valvular calcification [196]. In warfarin-induced uremic rats, a high dosage of Vitamin K1 intake was required to reverse VCand improve the elasticity of blood vessels [197].

### Anti-inflammatory compounds

As mentioned, pathological oxidative stress both intracellularly and extracellularly can contribute to calcification through a variety of mechanism including apoptosis, differentiation of smooth muscle cells to osteoblast-like cells. Such oxidative stresses can be counteracted and neutralized by potent antioxidants which are either synthetic or naturally occurring. The mechanisms of action of most of these compounds are similar, primarily involving the scavenging of ROS, such as hydroxyl radicals (the most reactive form of ROS), or the prevention of their formation altogether. Of noteworthy is that ROS, due to their high reactivity, can also interrupt normal cell function by damaging DNA, lipids of cell membrane, proteins, etc. Therefore, considering the important role ROS plays in the initiation and progression of calcification, antioxidants could be regarded as effective treatment options [198].

Curcumin, a polyphenol from Curcuma longa and turmeric, has well-documented anti-inflammatory and antioxidant properties [199–201]. Hou et al. [17] demonstrated its dose-dependent inhibition of β-glycerophosphate-induced VSMC calcification, reducing ALP, BMP2, Osterix, and Runx2 levels while preventing apoptosis. Curcumin also suppressed ectopic calcium deposition and promoted tendon regeneration by downregulating inflammatory proteins [17]. In another study, it was shown that curcumin can suppress the ectopic calcium deposition in-vitro and in-vivo in tendon. Moreover, curcumin effectively contributed to tendon regeneration through downregulation of inflammatory proteins under stimulated condition [18]. Liu et al. employed curcumin as a crosslinking agent to fix acellular bovine pericardium in bioprosthetic valves, which effectively reduced calcification due to curcumin’s anti-inflammatory effects [202]. Zhou et al. [19] found curcumin inhibited aortic valve cell trans-differentiation, with RNA sequencing linking its effects to NF-κB, TNF, MAPK, and PI3K-AKT pathways. Overall, all these findings highlight the importance of chronic inflammation, and the potential antioxidant can offer in the treatment of not only calcification but also generally cardiovascular complication.

Apocynin, also referred to as dogbane, is a herbal medicine and has potent anti-inflammation activity as reported in several animal studies [203]. It inhibits Nox and decreases superoxide generation [20, 21, 203]. It was shown that Apocynin administration to diabetic rats with VC substantially alleviated arterial calcium deposition [20]. In vitro, it has been suggested that apocynin prevents calcification by down-regulation of bone forming proteins such as BMP-2 and runt-related transcription factor 2 (RUNX2) [21].

Diosgenin as another natural antioxidants, is extracted from fenugreek (Trigonella foenum-graecum). Besides its anti-inflammation activity, Diosgenin has been shown to have a high efficiency in reducing the level of lipid and glucose in circulation [204]. Diosgenin strongly reduced calcification in uremic rat which was attributed to high antioxidant levels, and in turn low lipid peroxidation, thereby increasing endothelial nitric oxide synthase (eNOS) activities [22, 23]. Vitamin E, as another strong antioxidant, as well as its derivatives has shown to prevent osteogenic trans-differentiation of VSMCs induced by oxidized LDL [205], increases antioxidant level in circulation, and reduces calcification in uremic rats [206].

Puerarin, another herbal medicine, is extracted from *Pueraria lobata*, bears multiple active sites of isoflavone glycoside and is used for treating acute ischemic stroke. Puerarin reduces calcification by lowering ROS, reduction of NF-κB, and down-regulation of BMP-2 IL-1β levels [24]. Likewise, resveratrol and its derivatives which have similar active sites and found mostly in grapes and wines, has demonstrated positive impact on prevention of calcification as they increase Klotho level which are known as important inhibitors [25, 207].

Quercetin is another potent anti-inflammatory compound which is mostly found in variety of fruits and vegetables [208]. Its protective effects on vasculature have been demonstrated in several in vitro and in vivo studies [209]. Quercetin prevented heat shock proteins and thus reduced calcification in vitro [210]. It prevented VC in warfarin-fed animal models by downregulating transglutaminase 2 and β-catenin, reducing ROS production [211]. In a uremic rat model, quercetin increased superoxide dismutase 2 (SOD2) levels, preventing calcification via the iNOS/MAPK pathway [26]. In vitro, quercetin protects VSMCs stimulated by ox-LDL- from differentiation by down regulation of BMP-2 and toll-like receptor (TLR) − 4 [212].

Ginsenosides are one of the major components of traditional herbal medicine which are classified into panaxadiols and panaxatriols and also have potent anti-inflammatory properties. Rb1, a most abundant component of panaxadiols has reported protective properties for vascular and kidney diseases, alleviated creatine and inflammatory cytokine levels in CKD patients [213]. Rb1 has shown to reduce calcium deposition in vitro and *in vivo. In vitro* results have indicated the protective role of Rb1 in calcium deposition. Rb1 has reduced calcium deposition as well as ALP activity and calcium concentration *in vivo.* This protective effect of Rb1 is attributed to downregulation of Wnt/β-catenin pathway through the activation of nuclear receptor PPAR-γ [27].

Spermidine, a naturally synthesized polyamine, have been found to have anti-inflammatory properties and provides protection against CVD, reduce reduces lipid accumulation to retard the progression of atherosclerosis [214]. Liu et al. indicated similar results in attenuating calcification in VSMC and arterial rings in vitro and in vivo respectively. Spermidine has shown to activate Sirtulin 1(SIRT1) which inhibits vascular calcification in vitro resulted in the downregulation of endoplasmic reticulum (ER) stress signaling components, such as activating transcription factor 4 (ATF4) and CCAAT/enhancer-binding protein homologous protein (CHOP) [28]. Clinical trials on the application of spermidine for vascular calcification has not been investigated yet.

*Dendrobium officinale* polysaccharide (DOP) is a valuable herbal medicine and has been shown to enhance humoral and cellular immunity, anticancer, anti-inflammatory, antioxidant, and antidiabetic effects [215]. DOP has shown to inhibit VC in vitro and *in vivo.* DOP significantly stimulated the activation of HMOX-1 and responsible for anti-apoptotic and anti-inflammatory functions. Moreover, DOP has shown a reduced expression of inflammatory markers such as NLRP3, NF-κB, and IL-1β in VSMCs [29].

Clinical trials on the effect of antioxidants are yet to be investigated to determine their treatment efficacy. Currently there is little information in the literature in this regard. Sodium thiosulfate (STS) which is regarded both as a calcium chelator and an antioxidant has been the subject of a number of clinical trials [216–219] which will be discussed in the following section (chelation therapy). The positive impact of STS has been indicated in the treatment of VC. However, it is still unclear whether this is attributed to its chelation, anti-oxidation or both properties. Another antioxidant cerium nitrate-silver sulfadiazine was topically administered in France to patients with calciphylaxis whose mechanisms is comparable to VC [220]. The positive outcome of the compound was indicated and attributed to ROS scavenging, prevention of infection, as well as its decalcification ability.

### Antagonists

ALP inhibitors (e.g., 1,2-diphenyl-2-(1 H-1,2,4-triazol-5-ylthio)athanone) have been found to to prevent VSMC calcification in-vitro [221]. Therefore, therapeutic approaches could be devised to reduce the activity of ALP while maintaining normal bone formation/resorption [222, 223]. It has been found that Sortilin (SORT1) is responsible for ALP loading into the matrix vesicles during VC in-vivo. Sortilin deficiency in mice successfully attenuatedVC, without affecting bone calcification [224]. A study in 2019 showed that SBI-425, an ALP inhibitor, prevents medial arterial calcification in CKD mice models, while maintaining normal skeletal mineralization [30]. To verify its activity, Opdebeeck et al. [225], later in 2021 studied different dosages of SBI-425 to treat adenine-fed CKD rat model of calcification. Low dosages failed to reduce calcification, while high doses reduced bone mineralization. Overall, further studies could be directed on the ALP inhibitors that can merely target calcification in the vasculature not the hard tissues.

SNF472, hexasodium salt of phytate, developed as an intravenously administered form of phytate, is a calcification inhibitor by binding to HAp crystals without chelating free calcium. In vitro results suggest that SNF472 binds easily to HAp and not to free calcium. SNF472 also reduces the levels of calcium deposition in rodent VSMCwithout inducing apoptosis and restoring expression of genes that maintain the contractile phenotype of these cells [31]. Clinical trials in phase 1 and 2 have also shown that SNF472 inhibited HAp crystal formation and deposition without chelating the circulating calcium and hypocalcaemia [226].

Denosumab is a human monoclonal antibody that can bind to and suppress human receptor activator of nuclear factor kappa-B ligand (RANKL), mimicking osteoprotegerin natural bone-protecting activities. It is currently in development. Clinical trials in patients with bone metastases in multiple myeloma or breast cancer found that an injection of denosumab caused immediate and prolonged inhibition of bone turnover indicators [227]. A study with human RANKL knock-in (huRANKL-KI) mice demonstrated that there was a reduction of aortic calcium deposits of prednisolone-treated huRANKL-KI mice by up to 50% when treated with denosumab, based on calcium measurement [32]. The potential effectiveness of denosumab on coronary and abdominal aortic calcification in the elderly female population with osteoporotic chronic renal disease is being assessed in a clinical trial undergoing in France. An important factor in VC is the TNAP iso-enzyme, which can inactivate pyrophosphate, creating sites for bone mineralization and an increase in phosphate ions. Trials are in underway to create an oral medication that may function as a TNAP inhibitor and slow the onset and progression of calcification [228]. These trials’ outcomes will provide more insight into how ALP affects the calcification of coronaryVSMCs.

### Chelation therapy

In chemistry, chelation is the bonding of molecules with metal ions including transition metals (e.g., Cu^2+^) and earth metals (e.g., Ca^2+^). The specific chemical structure and configuration of the chelating material allow the formation of a strong complex with the metal ion. Chelation is of outmost importance not only in biology, where a chelant can be employed for the treatment of heavy metal poisoning, but also in industry where its small amount can effectively suppress mineral deposition [39, 128, 129, 229]. For complexation with calcium, the chemical structure of the chelator typically contains acidic residues such as phosphoric or carboxylic acid groups.

#### Ethylenediaminetetraacetic Acid (EDTA)

Ethylenediaminetetraacetic acid (EDTA) is one of the most efficient calcium chelators. EDTA can effectively disintegrate and dissolve highly insoluble HAp. Its ability in HAp dissolution was found to be superior to citric acid, Fostex (a commercial phosphate-based chelator) [230], DTPA, and STS [231]. On this basis, EDTA may possess the potential to reverse calcification, and thus, despite not being approved by the Food and Drug Administration, it is still prescribed by some practitioners for vascular diseases and calcification [232]. However, clinical studies on the systemic administration of EDTA has not shown promising outcomes. The use of IV-administered EDTA therapy on 1708 patients with a history of MI showed a modest reduction in the risk of adverse cardiovascular events [233]. The study which is called the Trial to Assess Chelation Therapy (TACT) was not convincing enough according to the authors to suggest its routine application as a clinical treatment for those with previous MI. Another small study on the combined use of EDTA/tetracycline on human trial (77 patients) showed reduction in coronary artery calcium score [234]. However, 43% of the patients did not respond to the therapy. Additionally, the trial did not have a control group for comparison. Another study on 47 patients with the history of coronary artery disease (CAD) treated with EDTA did not show significant change in flow-mediated vasodilation compared to placebo [235].

**Fig. 9:**
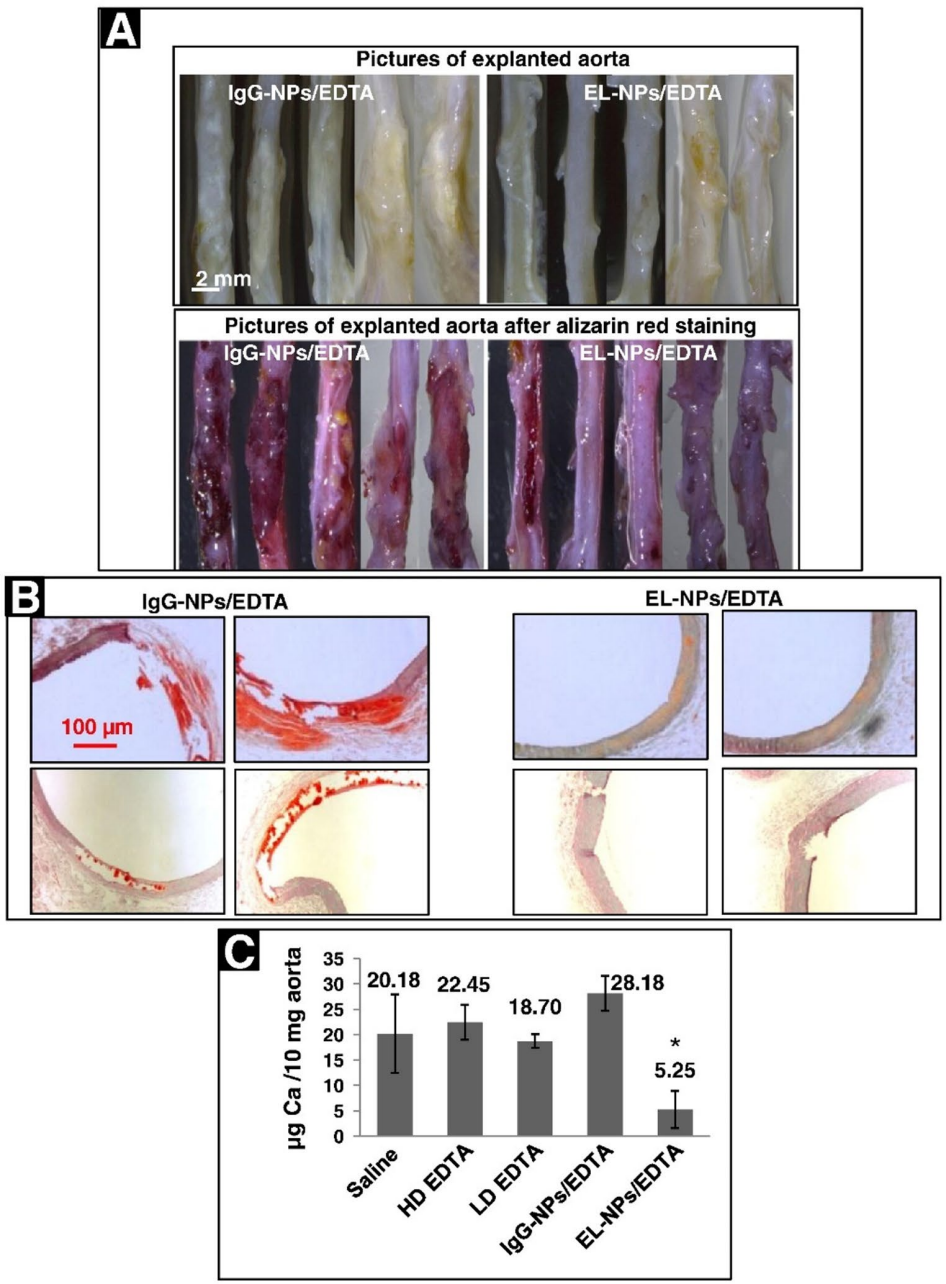
Treatment of vascular calcification by EDTA-loaded NPs. The NPs were conjugated with IgG or elastin antibody for targeting. (**A**) The explanted aortas were stained by Alizarin red to reveal calcium deposits in red colour. As seen the NPs coated with elastin antibody has a very less intense red colour, indicating efficacy of the NPs. (**B**) Histological staining by the same dye shows insignificant calcium in the aorta. (**C**) Quantification of calcium further confirms the efficiency of the targeting by elastin antibody

The considerable difference between human trial and dissolution ability of EDTA ex-vivo may be explained by several factors such as its low bioavailability in calcified plaque, its premature action upon injection, and its non-specificity (i.e., chelation other essential ions rather than calcium). Therefore, targeting EDTA to the calcified plaque could be an effective solution to address its low efficacy. EDTA was loaded into poly(lactide-co-glycolic acid) (PLGA) nanoparticles (NPs), which exhibited a sustained release within 3 days [231]. The NPs were delivered locally to a rat model with abdominal aortic calcification induced by CaCl_2_ injury. Calcification was reversed in the aorta while calcium and phosphate levels of the serum did not change. Another study loaded EDTA into albumin NPs onto which rabbit anti-rat elastin antibody was conjugated to target the calcified aortas [34]. The NPs were accumulated in the aorta significantly by conjugating the antibody, while in other organs (e.g., kidney, lung, and spleen), less NPs were detected. In-vivo studies on CaCl_2_ induced calcification showed efficacy of the prepared NPs on the regression of calcification as shown in Fig. 9. Targeted therapy had no effect on serum or urine calcium levels, while with systemic delivery, the former and the latter greatly decreased and increased, respectively. In a recent research, similar result were obtained with the same NPs on adenine-fed rat models of calcification [35].

#### Sodium thiosulfate (STS)

STS is an efficient chelating agent for transition metals as well as earth metal cations such as calcium. STS is a gold standard widely used for treating cyanide poisoning [236] and calciphylaxis (referred to as calcific uremic arteriolopathy) [237]. The latter involves calcification of small blood vessels in the fatty tissues and skin, which is often associated with renal failure and results in ulceration, infection, and death. STS has strong chelation properties, which let it sequester calcium from HAp, convert it to calcium thiosulfate, and excrete it from the body [231, 238]. Additionally, STS is known to have antioxidant properties [239]. Pasch et al. also studied the effects of STS on uremic adenine-fed rat models of calcification and found that STS inhibited calcification by lowering ionized calcium in plasma [36]. On 87 haemodialysis patients (IV injection, twice weekly post-haemodialysis for 4 months), STS inhibited the progression of coronary artery calcification. However, the bone mineral density of the total hip decreased [240]. Mathews et al. also indicated similar results in terms of inhibition of calcification (IV injection, 22 haemodialysis patients, 3 times a week for 5 months), however, no change in vertebral bone density was observed [219]. A recent study on 50 haemodialysis patients showed that intravenously-administered STS could effectively reduce the arterial stiffness [241]. However, bone mineral density was not studied. In summary, while STS is beneficial in inhibition of calcification, bone mineral density could be compromised, hindering its use clinically.

#### Bisphosphonates

**Fig. 10:**
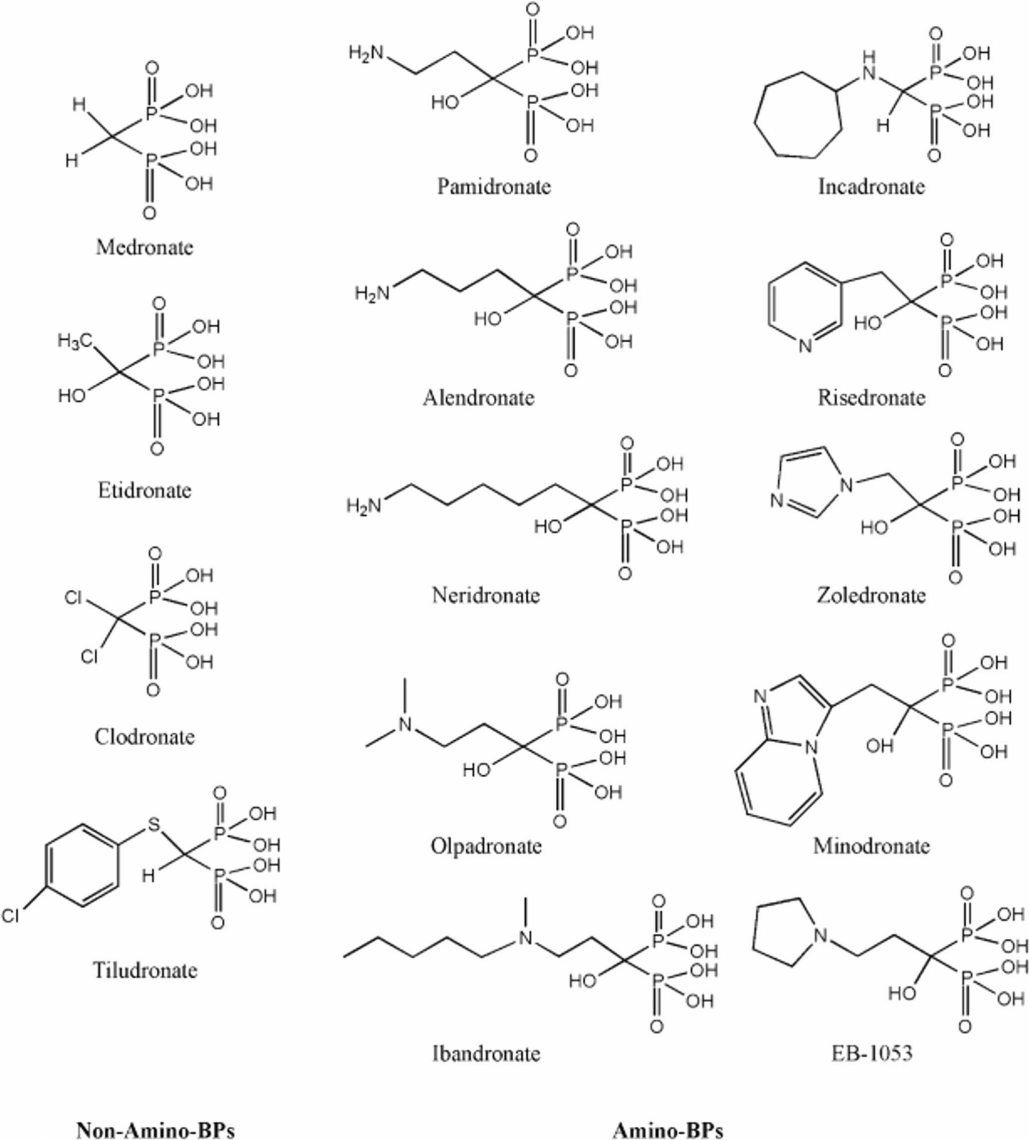
Chemical structure of bisphosphonates (nitrogen-free and nitrogen containing) employed in clinical treatment of bone diseases [243]

Bisphosphonates (BPs) or diphosphonates are a class of drug used for treating osteoporosis by suppression of bone resorption. Although their half-life in circulation is very short, when absorbed by bone, they can remain in the bone up to several years [242]. BPs are also called pyrophosphate analogous since the only difference is the central atom of the molecule, an oxygen in pyrophosphate and a carbon in BPs. Based on chemical structure as shown in Fig. 10, there are two types of BPs: nitrogen-containing (e.g. pamidronic acid) and nitrogen-free (etidronic acid).

In general, BPs have a strong affinity for HAp and calcium. Accordingly, a dual role has been proposed for BPs; they bind to the growing HAp crystals, preventing its further growth. On the other hand, the attached BPs on the HAp surface protect it from dissolution [242, 243]. In adenine-fed rat models of CKD, both types of BPs prevented aortic calcification but did not reverse it [37]. A low dosage of BP therapy did not adversely affect serum calcium or phosphorus levels despite preventing calcification. The inhibitory role of BPs thus appears to originate largely from inhibiting HAp nucleation/growth, rather than treating hypercalcemia or hyperphosphatemia. In other models of CKD, such as vitamin D_3_ [244], and warfarin-treated rats [38], BPs have prevented calcification.

While BPs have shown promising results in animal models, there is still no convincing human study suggesting their efficacy for calcification. The use of nitrogen-free etidronate (200 mg/day, orally, for 14 days, 3 times every 3 months) on 35 CKD patients prevented calcification (26/35 responded positively) [245]. However, another study on 42 CKD patients with the nitrogen-containing alendronate (18 months, 70 mg/week orally) did not reveal any significant positive outcome [246]. In addition, certain BPs may have adverse effects on the renal function when taken in high doses. For instance, nitrogen-containing pamidronate at a dosage of higher than 90 mg/month intravenously caused renal toxicity [247]. Of noteworthy is that BPs are more effective when administered intravenously rather than orally as observed in the treatment of osteoporosis [248]. The latter delivery route may also lead to gastrointestinal toxicity. Oral administration of BPs was not effective in the attenuation of calcification in patients with CKD and kidney transplants [246, 249]. However, they can potentially contribute to regression of atherosclerotic plaque by lowering serum lipid, LDL, and increasing high density lipoproteins (HDL) level [250, 251].

#### Polysuccinimide (PSI)

**Fig. 11:**
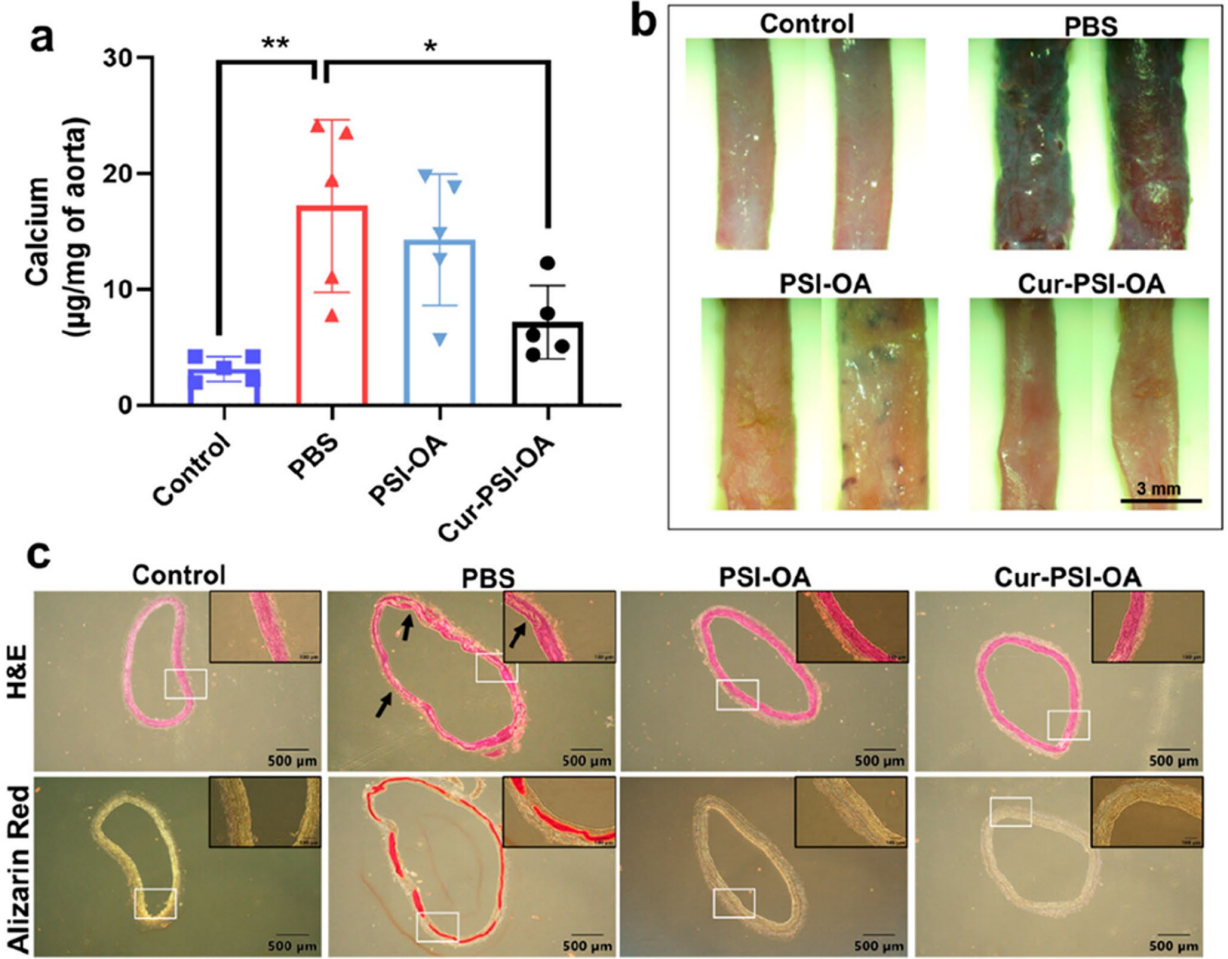
Treatment of Curcumin loaded PSI OA resulting in reduced calcium levels. (**A**) Quantitative measurement of calcium showing resulting in reduced calcium deposition in vivo. (**B**) Alizarin red staining on aorta indicating reduced calcium expression in curcumin loaded PSI OA nanoparticles (**C**) Histological analysis of the aortas and staining with alizarin red and H&E harvested from animals indicating a reduced calcium deposition for curcumin loaded PSI OA nanoparticles [39]

PSI, a hydrophobic polymer, is a precursor of polyaspartic acid [252–254] and acts as a strong chelating agent for different metal ions [129, 255, 256]. Several studies have been performed confirming the effectiveness of polyaspartic acid for inhibition and dissolution of calcium oxalate by the chelation effect of polyaspartic acid [158, 159]. A recent study explored the anticalcification and anti -ROS activity of PSI modified with oleylamine nanoparticle which also serves as a carrier for curcumin for the treatment of vascular calcification [39]. The application of curcumin loaded PSI-oleylamine nanoparticles have shown reduced calcification in the aorta without adversely affecting bone integrity after performing in vivo and in vitro studies (Fig. 11).

Collectively, despite the promising positive effects of calcium chelators, and despite being prescribed by some practitioners, its wide clinical use is still limited due to its side effects especially on the bone integrity. Improving the bioavailability of the chelator in the vasculature, while reducing its accumulation in bone could effectively address this issue. Targeting the chelator to the vasculature by employing an appropriate antibody such as elastin antibody was shown to significantly improve the treatment efficacy while not adversely affecting the bone integrity. Furthermore, the efficacy of chelation therapy could be further enhanced through the development of the chelators that specifically chelate calcium, rather than non-specific complexation with other essential ions such as iron, and magnesium.

### Others

Melatonin is an important indoleamine produced by pineal gland which has anti-inflammatory, anti-cancer, and antioxidant activities [257]. Many studies report the role of melatonin in regulating inflammation, apoptosis, and oxidative stress [258]. Melatonin attenuates VCby regulating autophagy via the AMP-activated protein kinase/mammalian target of rapamycin/Unc-51-like kinase 1 (AMPK/mTOR/ULK1) signaling pathway. In vitro results have revealed that melatonin activated AMPK protein expression, inhibited mTOR expression, and reduced VSMC calcification [40]. The effect of melatonin on VC was only studied in vitro, and in vivo studies of the effect of melatonin are not reported yet.

Metformin is an anti-diabetic and anti-hyperglycaemic agent which inhibit complex I of the mitochondrial electron transport chain and contribute to a decrease in the cell energy charge, thereby activating AMPK signalling. Metformin inhibits hyperlipidaemia-associated VC through anti-ferroptosis effects in vitro and in vivo. Ferroptosis is form of regulated cell death that involves metabolic dysfunction resulting from iron-dependent excessive lipid peroxidation and also involved in VC. Metformin have shown to inhibit ferroptosis in VSMCs, as evidenced by the upregulation of the anti-ferroptosis factors Gpx4, GSH, and SLC7A11 concomitant with the downregulation of lipid peroxidation. Moreover, metformin enhances antioxidative capacity in VC by promoting translocation of Nrf2 from the cytoplasm to the nucleus, which in turn promote transcription of genes encoding antioxidant proteins. In vivo study have shown a decrease in calcium deposition in aortic tissue [41].

### Clinical trials of VC treatments

Various therapeutics for VC were tested for various diseases as enlisted in Table 2. Sodium thiosulfate (STS) is a chelating agent which has proved to be successful in preclinical studies of VCby acting as a chelating agent. By removing calcium from precipitated minerals, STS improves endothelial function and produces more soluble calcium thiosulfate [259, 260]. Sodium thiosulfate in majority of the clinical trials have shown a positive effect in the treatment of VC at phase 2 clinical trials. Coronary artery calcification (CAC) score is one important parameter which determines the progression of calcification. Clinical trials of STS for VC have shown a decreased CAC along with reduced inflammatory markers core correlating to an improvement of calcification. Some studies have shown a non-persistent in serum calcium levels throughout the study and few side effects such as anorexia, nausea and vomiting were observed in few subjects [240, 261, 262]. Magnesium was shown to inhibit phosphate-induced VC in vitro and in animal models and reduce the progression ofVC. However, clinical studies for magnesium in improvement of calcification have shown no significant change [193, 263]. Similarly EDTA -tetracycline have shown a significant decline in calcification progression during preclinical studies but its therapeutic effect hasn’t been proved in clinical studies yet as they are still in recruitment phase [234]. Bisphosphonates have also shown promising results in decreased calcification, improvement in BMD score for CKD patients and have completed phase 4 in 2009. Denosumab and Sevelamer carbonate though have shown promising results in the in vitro preclinical studies with reduced CAC score, and decreased level of calcification markers, clinical studies were terminated at phase 4 due to adverse events occurring during the entire study along with variation in calcification scores, phosphorous and Morbi-mortality during the fixed time frame [32, 186]. From the above observation, STS and bisphosphonates are potential lead candidates for further human studies and could potentially be approved by the regulatory agencies for human application. Tables 2 and 3 describes the therapeutics for VCin clinical trials and its current status.

**Table 2:** Clinical trials of VC treatments (those where trial registration ID was not stated)

Therapeutics	Study group	Treatment protocol	Observation indicators	Results	Reference/Publication year
STS	Hemodialysis secondary hyperparathyroidism patients	Control and treatment group had no limit in calcium intake, phosphate intake 800–1,000 mg/day. Treatment group given STS 0.18 g/kg in 100 ml saline three times a week for 6 months	Parathyroid hormone (PTH), calcium, phosphorus, and calcium-phosphorus, coronary artery calcification (CAC) score	Levels of C-RP and CAC scores were significantly decreased in the treatment group (354.72 ± 45.22) than control (489.54 ± 65.47) Decrease in proportion of skin pruritus, myasthenia, bone pain, insomnia	[261] Published in 2022
STS	Adult hyperparathyroidism patients	Control and treatment group both received conventional bicarbonate haemodylasis (HD) twice or three times per week (4 h per session). Dialysate calcium concentration (2.0 to 3.5 mEq/L). Treatment group given STS (12.5 g intravenously, 15–20 min after HD treatment twice a week for at least 4 months)	Coronary artery calcification (CAC) score, bone mineral density (BMD)	Increase in serum sodium and chloride, a decline in serum bicarbonate and calcium, significant decline in BMD in treatment groups. However, CAC score unchanged in treatment group (904 (range 307–3399), 1014 (range 317–2178)	[240] Published in 2010
STS	Haemodialysis (HD) patients with calcific uraemic arteriolopathy	Randomized control trial. Patients received conventional bicarbonate haemodylasis (HD) twice or three times per week (4 h per session). Dialysate calcium concentration (1.25 to 1.5 mmol/L). Treatment group given 25 g/1.73 m^2^ (area of aorta based on abdominal aorta agatson calcification score) dissolved in 100 mL saline intravenously during the last 15 min	Abdominal aortic calcification score (AACS)	Parathyroid hormone significantly increased in treatment group (189 (69.1–403.8) vs. 129.7 (59.8–256.1). AACS significantly increased after 6 months of therapy (4823 vs. 3879)	[262] Published in 2020
Magnesium	CKD patients	Double-blinded placebo-controlled multicenter clinical trial. Treatment group provided with 52 weeks of treatment with oral slow-release MgOH twice daily (30 mmol of Mg per day)	Coronary artery calcification (CAC) score	No significant change in CAC between treatment and control placebo group. No effect in plasma phosphate, parathyroid, and potassium levels in plasma.	[263] Published in 2023
Magnesium	CKD patients	Double-blinded placebo-controlled multicenter clinical trial. Treatment group provided with 52 weeks of treatment with either oral slow-release Mg OH twice daily (30 mmol of Mg per day)	CAC score, bone mineral density (BMD)	Randomized control trials on human subjects have shown magnesium reduces coronary artery calcification after 52 week study	[193] Published in 2017
Vitamin K12	End stage kidney disease patients on haemodialysis	200 µg of vitamin 12 orally everyday for 1 year	Calcification of the abdominal aorta, MGP	Decrease in MGP by 53% in treatment group after 3 months. Calcification score showed no significant difference	[264] Published in 2019
(EDTA)- tetracyline	Coronary atherosclerotic heart disease patient	EDTA (1500 mg)- tetracycline (500 mg) taken orally once a week for 4 months	Coronary artery calcification (CAC) score, hs-CRP, Complete Blood Count (CBC), Metabolic Panel (CMP), Liver Functions Tests (LFT), Lipid Profile (LP), and NB ELISA antigen and NB IgG antibody serology	A significant decrease by 57% in CAC scores (1753.8 vs. 2033), improvement in lipid profile with a decrease in LDL (2.6 mmol/l vs. 2.1 mmol/l), triglycerides (2.5 mmol/l vs. 1.9 mmol/l), cholesterol (4.9 mmol/l vs. 4.2 mmol/l), and increase HDL	[234] Published in 2004
Bisphosphonates (alendronate)	Patients with CKD	70 mg alendronate administered orally once weekly for 18 months	Pulse wave velocity (PWV), BMD, Biochemical parametersC RP, and lipid profile	Increase in BMD of lumbar spine (0.84 ± 1.38 vs. 0.54 ± 1.89) and better PWV, no significant difference in kidney function however no significant difference in VCprogression between placebo and treatment	[246] Published in 2010
Denosumab	Patients with calcific aortic stenosis	Denosumab 60 mg every 6 months, placebo injection every 6 months, oral alendronic acid 70 mg once weekly. Trial end points after 12 and 24 months	Aortic valve calcium scoring, biochemical parameters	.	[265] Published in 2021

**Table 3:** Current progress of the clinical trials of vctreatments (those found from clinical trial Database)

Therapeutic	Study type and phase	Study group	Treatment protocol	Results	Side effects/limitations	Clinical trial reference
STS	Interventional Completed Phase 2	18 years and older (Adult, older Adult) coronary calcification on hemodialysis (*n* = 50) Inclusion Criteria: CAC score > 300 Life expectancy > 6 months Dialysis vintage > 6 months Exclusion Criteria: Non-compliance to hemodialysis	Drug: 25% intravenous (IV) sodium thiosulfate 50 ml IV drip twice/week post hemodialysis	High serum phosphate, calcium containing phosphate binder associated with increasing CAC. intravenous STS reduced the calcium burden in calcific uremic arteriopathy and soft tissue calcification		ClinicalTrials.gov ID NCT00720772 Published in 2009
STS	Interventional Terminated at Phase 3 due to Company decision	18 Years and older (Adult, older Adult) Calcific Uremic Arteriolopathy (*n* = 40)	Drug: Sodium thiosulfate at 25 g in 100 ml normal sterile saline for 28 days treatment phase Drug: Placebo	Not provided		ClinicalTrials.gov ID NCT02527213 Published in 2017
Magnesium	Interventional Completed Phase 2	18 Years and older with Chronic Kidney Disease (*n* = 250) Inclusion criteria: Glomerular filtration rate between 45 and 15 mL/min for > 3 months Serum total magnesium < 0,82 mmol/L and serum phosphate > 1,15 mmol/L Exclusion criteria: Kidney donor recipient, Parathyroid hormone > 600 ρmol/L, active malignancy	Dietary Supplement: Mablet 360 mg twice daily for 12 months Dietary Supplement: Placebo twice daily for 12 months.	Magnesium supplementation reduced VCin CKD by increasing calcium/phosphate solubility in serum, by inhibiting calcium influx into VSMC, by inhibiting intracellular pro-calcification enzymes in VSMC and by increasing activity of intracellular anti-calcification enzymes in VSMC		ClinicalTrials.gov ID NCT02542319 Published in 2022
Magnesium	Interventional (completed) Phase: Not Applicable	18 Years to 111 Years calcinosis patients Inclusion criteria: Male or female hemodialysis patients ≥ 18 years, HCO3- in venous plasma ≤ 23 mmol/L Exclusion criteria: Pregnant or lactating subjects, History of alcohol abuse, illicit drug use	Receives MgCl2 first, MgCl2 and Bicarbonate in second phase for 7 weeks	Results not provided		ClinicalTrials.gov ID NCT02621762 Published in 2017
Warfarin Rivaroxabam	Interventional Completed Phase 3	18 Years to 80 Years Coronary arterial fibrillation patients (*n* = 50)	Rivaroxaban 15 MG once daily orally + Dual anti-platelet therapy (clopidogrel (75 mg daily, orally) + aspirin (80 mg once daily, orally)) Warfarin (to reach an INR goal of 2-2.5) + Dual anti-platelet therapy (clopidogrel (75 mg daily, orally) + aspirin (80 mg once daily, orally))	Resolution of left ventricular thrombus at 3 months, the proportion of patients with adjudicated stroke and systemic emboli		ClinicalTrials.gov ID NCT05705089 Published in 2023
EDTA- tetracyline	Observational Currently in recruitment phase	18 Years to 80 Years ischemic heart disease (IHD) (*n* = 200)	Diagnostic Test: Blood samples Diagnostic Test: Collection of right atrial appendage tissue sample			ClinicalTrials.gov ID NCT04533282 Estimated completion 2025
Bisphosphonate (Alendronate)	Interventional Completed phase 4	18 Years to 85 Years CKD(*n* = 50) Inclusion criteria: Subjects with CKD Stage 3 (GFR between 30 and 59 ml/min) Subjects 18 years of age or older Exclusion: Pregnant, Subjects already taking bisphosphonates	Drug: Alendronate 70 mg weekly orally for 18 months Drug: Placebo weekly orally	Change in degree of arterial stiffness, VC of superficial femoral artery and aorta, bone mineral density, serum calcium and phosphate levels	Symptoms and severity of side effects from alendronate, Episodes of hypocalcemia	ClinicalTrials.gov ID NCT00395382 published 2009
Denosumab	Interventional Terminated phase 4 due to modified study	65 Years to 95 Years CKD Inclusion CKD stage 5 patient, Patient with osteoporosis Exclusion: Patient with a cancer or myeloma, HIV	Denosumab Patients receive subcutaneous injection of Denosumab (60 mg) every 6 months for 24 months NaCl (0.9%, 1 ml) (placebo) Patients receive a subcutaneous injection of NaCl every 6 months for 24 months	Relative variation of femoral bone mineral density after 24 months of follow-up. Relative variation of lumbar bone mineral density, coronary calcification scores after 24 months of follow-up	Adverse events occuring during the entire study, Relative variation of coronary calcification, abdominal aorta calcification scores after 24 months	ClinicalTrials.gov ID NCT02792413 Published in 2023
Denosumab	Double-Blind Randomized Controlled Trial	Patients > 50 years of age with calcific aortic stenosis Inclusion: Patients > 50 years with peak aortic jet velocity > 2.5 m/s and grade 2 to 4 aortic valve calcification Exclusion: Aortic valve surgery in the next 6 months; life expectancy < 2 years; inability to undergo scanning; treatment for osteoporosis with bisphosphonates or denosumab	Denosumab 60 mg every 6 months, placebo injection every 6 months, oral alendronic acid 70 mg once weekly. Trial end points after 12 and 24 months	Reduced levels of Serum C-terminal telopeptide by 50% ( 0.1 mcg/l vs. 0.25 mcg/l) but no difference in calcium score between treatment and control		[265] ClinicalTrials.gov ID NCT02132026 Published in 2021
Sevelamer carbonate	Interventional Terminated stage 4 due to low enrolment	18 Years and older CKD patients Inclusion: CKD stage 3 or 4, Serum intact PTH < 500 pg/mL Exclusion: HIV positive or AIDS, Pregnant or breastfeeding, MI within the last 6 months	Sevelamer carbonate 1,600 mg three times daily with meals for 12 weeks	The primary outcome measure will be the change in fibroblast growth factor FGF-23 concentrations	Change in VCbiomarker levels, endothelial dysfunction biomarker levels, inflammatory biomarker levels [Time frame: 12 weeks]	ClinicalTrials.gov ID NCT01277497 Published 2016
Sevelamer carbonate	Randomized, prospective, open-label, parallel group study	Fifty patients with CKD (stages 3 and 4) Inclusion: Age more than 18 years, CKD stage 3 or 4 (eGFR 15–60 ml/min/1.73 m^2^), not expected to start dialysis for 8 months, serum intact PTH less than 500 pg/ml Exclusion: Patients with diabetes, taking oral steroids, chemotherapy, and radiotherapy, and children below the age of 16 years	Treatment groups treated with SC 1600 mg three times a day for 12 weeks by oral route	Sevelamer treatment resulted in a significant decrease in levels of FGF-23, calcidiol, and calcitriol, whereas FGF-23 and calcitriol remained unchanged, significantly reduced the inflammatory markers IL-6, IL-8, IL-10, CRP, TNFα, and IFN-γ	Limited baselines parameters due to discontinuation of phosphate binder therapy in study subjects, who had stopped receiving it three weeks before starting the current study medications as part of a washout period	[186] ClinicalTrials.gov ID NCT01277497 Published 2021

## Conclusion

VC refers to the ectopic deposition of HAp in the vasculature, leading to a high rate of cardiovascular mortality, especially in patients with kidney diseases. This paper reviews VC in terms of mechanism, inducers, inhibitors, and the treatments examined in vivo, in vitro, and in clinical studies. Although a considerable effort has been devoted to seeking and developing an efficacious treatment, none has yet been established due to the complex and diverse characteristics of the disease. Nevertheless, several studies have successfully identified the role of important players, which further contributes to understanding the disease pathology, thereby paving the way for advancing the treatment efficacy. For example, chronic inflammation and its characteristic biomarkers such as IL-6 have been suggested as a promising target.

A plethora of studies has indicated the utmost importance of inflammation as it contributes to the initiation and progression of calcification. Aside from apoptosis, inflammation results in the osteogenic trans-differentiation of VSMCs, upregulatingALP, the most considerable promoter of calcification. Therapeutic approaches, including phosphate binders, magnesium, vitamin K supplementation, antioxidants, chelation therapy with compounds like EDTA and STS, as well as inhibitors such as bisphosphonates and denosumab, offer potential strategies for addressingVC. Furthermore, ongoing research on novel treatments, including melatonin, metformin, and other interventions, suggests a promising avenue for future therapeutic development. In addition to the therapeutic options that target inflammation, the positive role of calcium chelators has also been indicated as they can greatly alleviate oxidative stresses by reducing the calcium activity for apoptosis and salt deposition. Clinical trials of few lead therapeutics have shown potential applications for VCsuch as STS and bisphosphonates with minimal side effects. However, regulatory approval needs to be provided by regulatory agencies for human application.

In summary, understanding the mechanisms and potential treatment modalities for VC underscores the need for comprehensive approaches that target both the underlying pathological processes and the intricate regulatory pathways involved. Further research and clinical trials are warranted to validate and optimize these potential treatments, paving the way for more effective management and prevention of VC in various clinical contexts.

## Data Availability

Not applicable.

## Abbreviations

2-APB: 2-aminoethyl diphenylborinate
ALP: Alkaline phosphatase
AMPk: AMP-activated protein kinase
Apo E: Apolipoprotein E
BGP: β-glycerophosphate
BMD: Bone mineral density
BMP: Bone morphogenetic proteins
BP: Bisphosphonates
BVSMC: Bovine vascular smooth muscle cells
CAC: Coronary artery calcification
CKD: Chronic kidney disease
CRP: C reactive protein
CVD: Cardiovascular diseases
DOP: Dendrobium officinale polysaccharide
DTPA: Diethylenetriaminepentaacetic acid
EDTA: Ethylenediaminetetraacetic acid
ESRF: End stage renal failure
FGF: Fibroblast growth factor
GSH: Glutathione
HAp: Hydroxyapatite
HDL: High density lipoproteins
HIFs: Hypoxia-inducible factors
IFN: Interferon
IL: Interleukin
JNK: jun N-terminal kinases
LDL: Low density lipoproteins
MAPK: Mitogen-activated protein kinase
MGP: Matrix-Gla protein
MI: Myocardial infarction
MIP-1α: Macrophage inflammatory protein-1 alpha
MMP: Matrix metalloproteinase
MOVAS: Mouse aortic vascular smooth muscle cells
NPs: Nanoparticles
NTP: Nucleoside triphosphate pyrophosphohydrolase
OPN: Osteopontin
PLGA: Poly(lactic-co-glycolic acid)
PPAR-γ: Peroxisome proliferator-activated receptor-γ
PPi: Pyrophosphates
PSI: Poly succinimide
RANKL: Receptor activator of nuclear factor kappa-B ligand
ROS: Reactive oxygen species
Runx 2: Runt-related transcription factor 2
SGK1: Glucocorticoid-inducible kinase 1
SMA: Smooth muscle actin
STS: Sodium thiosulfate
TIMPs: Tissue inhibitors metalloproteinases
TNAP: Tissue non-specific alkaline phosphatase
TNF α: Tumour necrosis factor-α
TRPM 7: Transient receptor potential melastatin 7
VC: Vascular calcification
VSMC: Vascular smooth muscle cells

## References

[1] Strauss HW et al (2019) Vascular calcification: the evolving relationship of vascular calcification to major acute coronary events. J Nucl Med 60(9):1207–1212 31350320 10.2967/jnumed.119.230276

[2] Kristensen AT, Jakobsen JC, Olsen NT (2022) Percutaneous coronary intervention in calcified stenoses: a protocol for a systematic review with meta-analysis, trial sequential analysis and network meta-analysis. BMJ Open 12(9):e063884 10.1136/bmjopen-2022-063884 PMC10439345 36691161

[3] London GM et al (2003) Arterial media calcification in end-stage renal disease: impact on all-cause and cardiovascular mortality. Nephrol Dialysis Transplantation 18(9):1731–1740 10.1093/ndt/gfg414 12937218

[4] Russo D et al (2011) Progression of coronary artery calcification and cardiac events in patients with chronic renal disease not receiving Dialysis. Kidney Int 80(1):112–118 21451461 10.1038/ki.2011.69 PMC3257039

[5] Cozzolino M et al (2018) Cardiovascular disease in Dialysis patients. Nephrol Dialysis Transplantation 33(suppl3):iii28–iii34 10.1093/ndt/gfy174 PMC6168816 30281132

[6] Modi ZJ et al (2019) Risk of cardiovascular disease and mortality in young adults with end-stage renal disease: an analysis of the US renal data system. JAMA Cardiol 4(4):353–362 30892557 10.1001/jamacardio.2019.0375 PMC6484951

[7] Fuery MA et al (2018) Vascular ossification: Pathology, mechanisms, and clinical implications. Bone 109:28–34 28688892 10.1016/j.bone.2017.07.006

[8] Bertazzo S et al (2013) Nano-analytical electron microscopy reveals fundamental insights into human cardiovascular tissue calcification. Nat Mater 12(6):576 23603848 10.1038/nmat3627 PMC5833942

[9] Singh A, Tandon S, Tandon C (2021) An update on vascular calcification and potential therapeutics. Mol Biol Rep 48(1):887–896 33394226 10.1007/s11033-020-06086-y

[10] Ren S-C et al (2022) Vascular calcification in chronic kidney disease: an update and perspective. Aging Disease 13(3):673 35656113 10.14336/AD.2021.1024 PMC9116919

[11] Wu M, Rementer C, Giachelli CM (2013) Vascular calcification: an update on mechanisms and challenges in treatment. Calcif Tissue Int 93:365–373 23456027 10.1007/s00223-013-9712-z PMC3714357

[12] Montezano AC et al (2010) Vascular smooth muscle cell differentiation to an osteogenic phenotype involves TRPM7 modulation by magnesium. Hypertension 56(3):453–462 20696983 10.1161/HYPERTENSIONAHA.110.152058

[13] Louvet L et al (2012) Magnesium prevents phosphate-induced calcification in human aortic vascular smooth muscle cells. Nephrol Dialysis Transplantation 28(4):869–878 10.1093/ndt/gfs520 PMC3611891 23229924

[14] Van den Broek F, Beynen A (1998) The influence of dietary phosphorus and magnesium concentrations on the calcium content of heart and kidneys of DBA/2 and NMRI mice. Lab Anim 32(4):483–491 9807763 10.1258/002367798780599758

[15] Pen J et al (2012) *The effect of the magnesium supplementation on vascular calcification in rats.* Zhongguo ying yong sheng li xue za zhi = Zhongguo yingyong shenglixue zazhi = Chinese. J Appl Physiol 28(1):20–23 22493887

[16] Yao Z et al (2018) Magnesium citrate protects against vascular calcification in an Adenine-induced chronic renal failure rat model. J Cardiovasc Pharmacol 72(6):270–276 29738375 10.1097/FJC.0000000000000590

[17] Hou M et al (2016) Curcumin attenuates osteogenic differentiation and calcification of rat vascular smooth muscle cells. Mol Cell Biochem 420(1):151–160 27502306 10.1007/s11010-016-2778-y

[18] Chen Y et al (2019) Controlled-release Curcumin attenuates progression of tendon ectopic calcification by regulating the differentiation of tendon stem/progenitor cells. Mater Sci Engineering: C 103:109711 10.1016/j.msec.2019.04.090 31349489

[19] Zhou T et al (2020) Curcumin inhibits calcification of human aortic valve interstitial cells by interfering NF-κB, AKT, and ERK pathways. Phytother Res 34(8):2074–2081 32189385 10.1002/ptr.6674

[20] Brodeur MR et al (2014) Reduction of advanced-glycation end products levels and Inhibition of RAGE signaling decreases rat vascular calcification induced by diabetes. PLoS ONE 9(1):e85922 24465790 10.1371/journal.pone.0085922 PMC3897559

[21] Feng W et al (2016) Apocynin attenuates angiotensin II-induced vascular smooth muscle cells osteogenic switching via suppressing extracellular signal-regulated kinase 1/2. Oncotarget 7(50):83588 27835878 10.18632/oncotarget.13193 PMC5347790

[22] Manivannan J et al (2013) Diosgenin attenuates vascular calcification in chronic renal failure rats. Mol Cell Biochem 378(1):9–18 23423339 10.1007/s11010-013-1588-8

[23] Manivannan J et al (2014) Diosgenin interferes coronary vasoconstriction and inhibits osteochondrogenic transdifferentiation of aortic VSMC in CRF rats. Biochimie 102:183–187 24742379 10.1016/j.biochi.2014.03.011

[24] Liu H et al (2019) Puerarin inhibits vascular calcification of uremic rats. Eur J Pharmacol 855:235–243 31085237 10.1016/j.ejphar.2019.05.023

[25] Zhang P et al (2016) Resveratrol ameliorated vascular calcification by regulating Sirt-1 and Nrf2. In: Transplantation proceedings. Elsevier 10.1016/j.transproceed.2016.10.023 27931585

[26] Chang X-y et al (2017) Quercetin attenuates vascular calcification through suppressed oxidative stress in adenine-induced chronic renal failure rats. BioMed Res Int 2017 10.1155/2017/5716204 PMC5485304 28691026

[27] Zhou P et al (2019) Ginsenoside Rb1 ameliorates CKD-associated vascular calcification by inhibiting the Wnt/β‐catenin pathway. J Cell Mol Med 23(10):7088–7098 31423730 10.1111/jcmm.14611 PMC6787443

[28] Liu X et al (2021) Spermidine inhibits vascular calcification in chronic kidney disease through modulation of SIRT1 signaling pathway. Aging Cell 20(6):e13377 33969611 10.1111/acel.13377 PMC8208796

[29] Jiang W, Ruan W, Wang Z (2022) Dendrobium officinale polysaccharide inhibits vascular calcification via anti-inflammatory and anti‐apoptotic effects in chronic kidney disease. FASEB J 36(9):e22504 35980507 10.1096/fj.202200353RRR

[30] Tani T et al (2020) Inhibition of tissue-nonspecific alkaline phosphatase protects against medial arterial calcification and improves survival probability in the CKD‐MBD mouse model. J Pathol 250(1):30–41 31509234 10.1002/path.5346 PMC7238767

[31] Perelló J et al (2020) Mechanism of action of SNF472, a novel calcification inhibitor to treat vascular calcification and calciphylaxis. Br J Pharmacol 177(19):4400–4415 32557649 10.1111/bph.15163 PMC7484563

[32] Helas S et al (2009) Inhibition of receptor activator of NF-κB ligand by denosumab attenuates vascular calcium deposition in mice. Am J Pathol 175(2):473–478 19590040 10.2353/ajpath.2009.080957 PMC2716948

[33] Lei Y et al (2013) Efficacy of reversal of aortic calcification by chelating agents. Calcif Tissue Int 93:426–435 23963635 10.1007/s00223-013-9780-0 PMC3809012

[34] Lei Y, Nosoudi N, Vyavahare N (2014) Targeted chelation therapy with EDTA-loaded albumin nanoparticles regresses arterial calcification without causing systemic side effects. J Controlled Release 196:79–86 10.1016/j.jconrel.2014.09.029 PMC4268131 25285609

[35] Karamched SR et al (2019) Site-specific chelation therapy with EDTA-loaded albumin nanoparticles reverses arterial calcification in a rat model of chronic kidney disease. Sci Rep 9(1):2629 30796300 10.1038/s41598-019-39639-8 PMC6385348

[36] Pasch A et al (2008) Sodium thiosulfate prevents vascular calcifications in uremic rats. Kidney Int 74(11):1444–1453 18818688 10.1038/ki.2008.455

[37] Lomashvili KA et al (2009) Effect of bisphosphonates on vascular calcification and bone metabolism in experimental renal failure. Kidney Int 75(6):617–625 19129793 10.1038/ki.2008.646 PMC2999577

[38] Price PA, Faus SA, Williamson MK (2001) Bisphosphonates alendronate and ibandronate inhibit artery calcification at doses comparable to those that inhibit bone resorption. Arterioscler Thromb Vasc Biol 21(5):817–824 11348880 10.1161/01.atv.21.5.817

[39] Adelnia H et al (2023) A bioactive disintegrable polymer nanoparticle for synergistic vascular anticalcification. ACS Nano 17(19):18775–18791 37650798 10.1021/acsnano.3c03041

[40] Chen WR et al (2020) Melatonin attenuates vascular calcification by activating autophagy via an AMPK/mTOR/ULK1 signaling pathway. Exp Cell Res 389(1):111883 32014443 10.1016/j.yexcr.2020.111883

[41] Ma W-Q et al (2021) Metformin attenuates hyperlipidaemia-associated vascular calcification through anti-ferroptotic effects. Free Radic Biol Med 165:229–242 33513420 10.1016/j.freeradbiomed.2021.01.033

[42] Tintut Y et al (2000) Tumor necrosis factor-α promotes in vitro calcification of vascular cells via the cAMP pathway. Circulation 102(21):2636–2642 11085968 10.1161/01.cir.102.21.2636

[43] Otsuka F et al (2014) Has our Understanding of calcification in human coronary atherosclerosis progressed? Thromb Vascular Biology 34(4):724–736Arteriosclerosis 10.1161/ATVBAHA.113.302642 PMC4095985 24558104

[44] Krohn JB et al (2016) Extracellular vesicles in cardiovascular calcification: expanding current paradigms. J Physiol 594(11):2895–2903 26824781 10.1113/JP271338 PMC4887674

[45] Giachelli C (2005) Inducers and inhibitors of biomineralization: lessons from pathological calcification. Orthod Craniofac Res 8(4):229–231 16238602 10.1111/j.1601-6343.2005.00345.x

[46] Naganuma T et al (2019) Hypercalcemia is a risk factor for the progression of aortic calcification in kidney transplant recipients. Kidney Blood Press Res 44(4):823–834 31266041 10.1159/000501740

[47] Conigrave AD, van Oostwaard MM (2019) Calcium disorders. Advanced practice in endocrinology nursing. Springer, pp 975–987

[48] Koumakis E et al (2021) The causes of hypo-and hyperphosphatemia in humans. Calcif Tissue Int 108(1):41–73 32285168 10.1007/s00223-020-00664-9

[49] Bazydlo LA, Needham M, Harris NS (2014) Calcium, magnesium, and phosphate. Lab Med 45(1):e44–e50

[50] Lian IA, Åsberg A (2018) Should total calcium be adjusted for albumin? A retrospective observational study of laboratory data from central Norway. BMJ Open 8(4):e017703 29627804 10.1136/bmjopen-2017-017703 PMC5892769

[51] Reynolds JL et al (2004) Human vascular smooth muscle cells undergo vesicle-mediated calcification in response to changes in extracellular calcium and phosphate concentrations: a potential mechanism for accelerated vascular calcification in ESRD. J Am Soc Nephrol 15(11):2857–2867 15504939 10.1097/01.ASN.0000141960.01035.28

[52] Schantl AE et al (2020) Inhibition of vascular calcification by inositol phosphates derivatized with ethylene glycol oligomers. Nat Commun 11(1):1–17 32024848 10.1038/s41467-019-14091-4 PMC7002685

[53] Kuro-o M (2013) Klotho, phosphate and FGF-23 in ageing and disturbed mineral metabolism. Nat Rev Nephrol 9(11):650–660 23774819 10.1038/nrneph.2013.111

[54] Pasch A et al (2012) Nanoparticle-based test measures overall propensity for calcification in serum. J Am Soc Nephrol 23(10):1744–1752 22956818 10.1681/ASN.2012030240 PMC3458464

[55] Chen W et al (2019) Patients with advanced chronic kidney disease and vascular calcification have a large hydrodynamic radius of secondary calciprotein particles. Nephrol Dialysis Transplantation 34(6):992–1000 10.1093/ndt/gfy117 PMC6545469 29788425

[56] Aghagolzadeh P et al (2016) Calcification of vascular smooth muscle cells is induced by secondary calciprotein particles and enhanced by tumor necrosis factor-α. Atherosclerosis 251:404–414 27289275 10.1016/j.atherosclerosis.2016.05.044

[57] Kendrick J et al (2018) Effect of treatment of metabolic acidosis on vascular endothelial function in patients with CKD: A pilot randomized cross-over study. Clin J Am Soc Nephrol 13(10):1463–1470 30237219 10.2215/CJN.00380118 PMC6218835

[58] Villa-Bellosta R, Gonzalez-Parra E, Egido J (2016) Alkalosis and dialytic clearance of phosphate increases phosphatase activity: a hidden consequence of Hemodialysis. PLoS ONE 11(7):e0159858 27454315 10.1371/journal.pone.0159858 PMC4959680

[59] Voelkl J et al (2019) Signaling pathways involved in vascular smooth muscle cell calcification during hyperphosphatemia. Cell Mol Life Sci 76(11):2077–2091 30887097 10.1007/s00018-019-03054-z PMC6502780

[60] Speer MY et al (2010) Runx2/Cbfa1, but not loss of myocardin, is required for smooth muscle cell lineage reprogramming toward osteochondrogenesis. J Cell Biochem 110(4):935–947 20564193 10.1002/jcb.22607 PMC2895022

[61] Lau WL et al (2011) Direct effects of phosphate on vascular cell function. Adv Chronic Kidney Dis 18(2):105–112 21406295 10.1053/j.ackd.2010.12.002 PMC3086393

[62] Chen NX et al (2002) Phosphorus and uremic serum up-regulate osteopontin expression in vascular smooth muscle cells. Kidney Int 62(5):1724–1731 12371973 10.1046/j.1523-1755.2002.00625.x

[63] Fakhry M et al (2017) TNAP stimulates vascular smooth muscle cell trans-differentiation into chondrocytes through calcium deposition and BMP-2 activation: possible implication in atherosclerotic plaque stability. Biochim Biophys Acta 1863(3):643–653 10.1016/j.bbadis.2016.12.003 27932058

[64] Poetsch F et al (2020) Role of SGK1 in the osteogenic transdifferentiation and calcification of vascular smooth muscle cells promoted by hyperglycemic conditions. Int J Mol Sci 21(19):7207 33003561 10.3390/ijms21197207 PMC7583813

[65] Bessueille L et al (2015) Glucose stimulates chondrocyte differentiation of vascular smooth muscle cells and calcification: A possible role for IL-1β. FEBS Lett 589(19):2797–2804 26277062 10.1016/j.febslet.2015.07.045

[66] Zhang P et al (2020) Apelin-13 attenuates high glucose-induced calcification of MOVAS cells by regulating MAPKs and PI3K/AKT pathways and ROS-mediated signals, vol 128. Biomed Pharmacother: 110271 10.1016/j.biopha.2020.110271 32450527

[67] Li G et al (2014) The relationship between serum hypoxia-inducible factor 1α and coronary artery calcification in asymptomatic type 2 diabetic patients. Cardiovasc Diabetol 13(1):1–8 24564828 10.1186/1475-2840-13-52 PMC3938975

[68] Ruffenach G et al (2016) Role for runt-related transcription factor 2 in proliferative and calcified vascular lesions in pulmonary arterial hypertension. Am J Respir Crit Care Med 194(10):1273–1285 27149112 10.1164/rccm.201512-2380OC

[69] Chatrou ML et al (2015) Intra-section analysis of human coronary arteries reveals a potential role for micro-calcifications in macrophage recruitment in the early stage of atherosclerosis. PLoS ONE 10(11):e0142335 26555788 10.1371/journal.pone.0142335 PMC4640818

[70] Holtz KM, Kantrowitz ER (1999) The mechanism of the alkaline phosphatase reaction: insights from NMR, crystallography and site-specific mutagenesis. FEBS Letter 462(1–2):7–11 10.1016/s0014-5793(99)01448-9 10580082

[71] Lomashvili K et al (2008) Upregulation of alkaline phosphatase and pyrophosphate hydrolysis: potential mechanism for uremic vascular calcification. Kidney Int 73(9):1024–1030 18288101 10.1038/ki.2008.26 PMC3010853

[72] SiLcox DC, McCarty DJ (1973) Measurement of inorganic pyrophosphate in biological fluids, elevated levels in some patients with osteoarthritis, pseudogout, acromegaly, and uremia. J Clin Investig 52(8):1863–1870 4352576 10.1172/JCI107369 PMC302467

[73] Narisawa S, Yadav MC, Millán JL (2013) Vivo overexpression of tissue-nonspecific alkaline phosphatase increases skeletal mineralization and affects the phosphorylation status of osteopontin. J Bone Miner Res 28(7):1587–1598 23427088 10.1002/jbmr.1901 PMC3688694

[74] Mazhar AR et al (2001) Risk factors and mortality associated with calciphylaxis in end-stage renal disease. Kidney Int 60(1):324–332 11422768 10.1046/j.1523-1755.2001.00803.x

[75] Lomashvili KA, Khawandi W, O’Neill WC (2005) Reduced plasma pyrophosphate levels in Hemodialysis patients. J Am Soc Nephrol 16(8):2495–2500 15958726 10.1681/ASN.2004080694

[76] Jono S, Peinado C, Giachelli CM (2000) Phosphorylation of osteopontin is required for Inhibition of vascular smooth muscle cell calcification. J Biol Chem 275(26):20197–20203 10766759 10.1074/jbc.M909174199

[77] Druck A et al (2019) Osteopontin levels in patients with chronic kidney disease stage 5 on Hemodialysis directly correlate with intact parathyroid hormone and alkaline phosphatase. Clin Appl Thromb Hemost 25:1076029619896621 31876180 10.1177/1076029619896621 PMC7019405

[78] Steitz SA et al (2001) Smooth muscle cell phenotypic transition associated with calcification: upregulation of Cbfa1 and downregulation of smooth muscle lineage markers. Circul Res 89(12):1147–1154 10.1161/hh2401.101070 11739279

[79] Shanahan CM et al (2011) Arterial calcification in chronic kidney disease: key roles for calcium and phosphate. Circul Res 109(6):697–711 10.1161/CIRCRESAHA.110.234914 PMC3249146 21885837

[80] Shobeiri N, Adams MA, Holden RM (2014) Phosphate: an old bone molecule but new cardiovascular risk factor. Br J Clin Pharmacol 77(1):39–54 23506202 10.1111/bcp.12117 PMC3895346

[81] Lee SJ, Lee I-K, Jeon J-H (2020) Vascular calcification—new insights into its mechanism. Int J Mol Sci 21(8):2685 32294899 10.3390/ijms21082685 PMC7216228

[82] Boraldi F, Lofaro FD, Quaglino D (2021) Apoptosis in the extraosseous calcification process. Cells 10(1):131 33445441 10.3390/cells10010131 PMC7827519

[83] Zheng KH et al (2019) Lipoprotein (a) and oxidized phospholipids promote valve calcification in patients with aortic stenosis. J Am Coll Cardiol 73(17):2150–2162 31047003 10.1016/j.jacc.2019.01.070 PMC6494952

[84] Chistiakov DA et al (2017) Calcifying matrix vesicles and atherosclerosis. BioMed Res Int, 2017 10.1155/2017/7463590 PMC5697392 29238720

[85] Hashimoto S et al (1998) Chondrocyte-derived apoptotic bodies and calcification of articular cartilage. Proc Natl Acad Sci 95(6):3094–3099 9501221 10.1073/pnas.95.6.3094 PMC19700

[86] Clarke MC et al (2008) Chronic apoptosis of vascular smooth muscle cells accelerates atherosclerosis and promotes calcification and medial degeneration. Circul Res 102(12):1529–1538 10.1161/CIRCRESAHA.108.175976 18497329

[87] Ciceri P et al (2016) Iron citrate reduces high phosphate-induced vascular calcification by inhibiting apoptosis. Atherosclerosis 254:93–101 27716569 10.1016/j.atherosclerosis.2016.09.071

[88] New SE, Aikawa E (2011) Cardiovascular calcification–an inflammatory disease–. Circ J: 1105101223–1105101223 10.1253/circj.cj-11-0395 21566338

[89] Kurozumi A et al (2019) IL-6 and sIL-6R induces STAT3-dependent differentiation of human VSMCs into osteoblast-like cells through JMJD2B-mediated histone demethylation of RUNX2. Bone 124:53–61 30981888 10.1016/j.bone.2019.04.006

[90] Chai S et al (2021) Positive association of leptin and artery calcification of lower extremity in patients with type 2 diabetes mellitus: a pilot study. Front Endocrinol 12 10.3389/fendo.2021.583575 PMC8170469 34093426

[91] Lee C-T et al (2013) Biomarkers associated with vascular and valvular calcification in chronic Hemodialysis patients. Dis Markers 34(4):229–235 23396289 10.3233/DMA-130965 PMC3810241

[92] Dervisoglu E et al (2008) Serum fetuin-a concentrations are inversely related to cytokine concentrations in patients with chronic renal failure. Cytokine 44(3):323–327 18922701 10.1016/j.cyto.2008.08.014

[93] Lim K et al (2012) Vascular Klotho deficiency potentiates the development of human artery calcification and mediates resistance to fibroblast growth factor 23. Circulation 125(18):2243–2255 22492635 10.1161/CIRCULATIONAHA.111.053405

[94] Lopez-Mejias R, Gonzalez-Gay MA (2019) IL-6: linking chronic inflammation and vascular calcification. Nat Rev Rheumatol 15(8):457–459 31235835 10.1038/s41584-019-0259-x

[95] Hénaut L, Massy ZA (2018) New insights into the key role of Interleukin 6 in vascular calcification of chronic kidney disease. Oxford University Press, pp 543–548 10.1093/ndt/gfx379 29420799

[96] Park H-K, Ahima RS (2015) Physiology of leptin: energy homeostasis, neuroendocrine function and metabolism. Metabolism 64(1):24–34 25199978 10.1016/j.metabol.2014.08.004 PMC4267898

[97] Wolf G et al (2002) Leptin and renal disease. Am J Kidney Dis 39(1):1–11 11774095 10.1053/ajkd.2002.29865

[98] Parhami F et al (2001) Leptin enhances the calcification of vascular cells: artery wall as a target of leptin. Circul Res 88(9):954–960 10.1161/hh0901.090975 11349006

[99] Yamagishi S-i et al (2001) Leptin induces mitochondrial superoxide production and monocyte chemoattractant protein-1 expression in aortic endothelial cells by increasing fatty acid oxidation via protein kinase A. J Biol Chem 276(27):25096–25100 11342529 10.1074/jbc.M007383200

[100] Sorescu GP et al (2003) Bone morphogenic protein 4 produced in endothelial cells by oscillatory shear stress stimulates an inflammatory response. J Biol Chem 278(33):31128–31135 12766166 10.1074/jbc.M300703200

[101] Holloway WR et al (2002) Leptin inhibits osteoclast generation. J Bone Miner Res 17(2):200–209 11811550 10.1359/jbmr.2002.17.2.200

[102] Zeadin M et al (2009) Effect of leptin on vascular calcification in Apolipoprotein E–deficient mice. Arterioscler Thromb Vasc Biol 29(12):2069–2075 19797706 10.1161/ATVBAHA.109.195255

[103] Szulc P et al (2018) Positive association of high leptin level and abdominal aortic calcification in Men―The prospective MINOS Study―. Circ J 82(12):2954–2961 30282882 10.1253/circj.CJ-18-0517

[104] Zittermann A (2003) Vitamin D in preventive medicine: are we ignoring the evidence? Br J Nutr 89(5):552–572 12720576 10.1079/BJN2003837

[105] Zehnder D et al (2002) Synthesis of 1, 25-dihydroxyvitamin D3 by human endothelial cells is regulated by inflammatory cytokines: a novel autocrine determinant of vascular cell adhesion. J Am Soc Nephrol 13(3):621–629 11856765 10.1681/ASN.V133621

[106] Shi Y et al (2018) Fibroblast growth factor 21 ameliorates vascular calcification by inhibiting osteogenic transition in vitamin D3 plus nicotine-treated rats. Biochem Biophys Res Commun 495(4):2448–2455 29273504 10.1016/j.bbrc.2017.10.115

[107] Zhang J et al (2015) Thyroid hormone attenuates vascular calcification induced by vitamin D3 plus nicotine in rats. Calcif Tissue Int 96(1):80–87 25416842 10.1007/s00223-014-9934-8

[108] Ellam T et al (2014) Vitamin D deficiency and exogenous vitamin D excess similarly increase diffuse atherosclerotic calcification in Apolipoprotein E knockout mice. PLoS ONE 9(2):e88767 24586387 10.1371/journal.pone.0088767 PMC3929524

[109] Zittermann A, Schleithoff SS, Koerfer R (2007) Vitamin D and vascular calcification. Curr Opin Lipidol 18(1):41–46 17218831 10.1097/MOL.0b013e328011c6fc

[110] Trion A et al (2008) Modulation of calcification of vascular smooth muscle cells in culture by calcium antagonists, statins, and their combination. Mol Cell Biochem 308(1):25–33 17909945 10.1007/s11010-007-9608-1 PMC2226060

[111] Saremi A et al (2012) Progression of vascular calcification is increased with Statin use in the veterans affairs diabetes trial (VADT). Diabetes Care 35(11):2390–2392 22875226 10.2337/dc12-0464 PMC3476911

[112] Henein M et al (2015) High dose and long-term Statin therapy accelerate coronary artery calcification. Int J Cardiol 184:581–586 25769003 10.1016/j.ijcard.2015.02.072

[113] Dykun I et al (2016) Statin medication enhances progression of coronary artery calcification: the heinz Nixdorf recall study. J Am Coll Cardiol 68(19):2123–2125 27810054 10.1016/j.jacc.2016.08.040

[114] Puri R et al (2015) Impact of Statins on serial coronary calcification during atheroma progression and regression. J Am Coll Cardiol 65(13):1273–1282 25835438 10.1016/j.jacc.2015.01.036

[115] Giachelli CM, Donato M, Scatena M (2024) Matrix metalloproteinase-3 joins a growing list of proteases that regulate vascular calcification. Oxford University Press UK, pp 565–566 10.1093/cvr/cvae064 PMC11074787 38630897

[116] Elahirad S et al (2022) Association of matrix metalloproteinase-2 (MMP-2) and MMP-9 promoter polymorphisms, their serum levels, and activities with coronary artery calcification (CAC) in an Iranian population. Cardiovasc Toxicol 22(2):118–129 34731407 10.1007/s12012-021-09707-5

[117] Liang X et al (2021) Inflammatory cells accelerated carotid artery calcification via MMP9: evidences from single-cell analysis. Front Cardiovasc Med 8:766613 34938784 10.3389/fcvm.2021.766613 PMC8685327

[118] Xie Y et al (2024) Smooth muscle cell-specific matrix metalloproteinase 3 deletion reduces osteogenic transformation and medial artery calcification. Cardiovascular Res 120(6):658–670 10.1093/cvr/cvae035 PMC11074797 38454645

[119] Atkinson G et al (2023) The contribution of matrix metalloproteinases and their inhibitors to the development, progression, and rupture of abdominal aortic aneurysms. Front Cardiovasc Med 10:1248561 37799778 10.3389/fcvm.2023.1248561 PMC10549934

[120] Ron D, Walter P (2007) Signal integration in the Endoplasmic reticulum unfolded protein response. Nat Rev Mol Cell Biol 8(7):519–529 17565364 10.1038/nrm2199

[121] Furmanik M et al (2021) Endoplasmic reticulum stress mediates vascular smooth muscle cell calcification via increased release of Grp78 (glucose-regulated protein, 78 kDa)-loaded extracellular vesicles. Arterioscler Thromb Vasc Biol 41(2):898–914 33297752 10.1161/ATVBAHA.120.315506 PMC7837691

[122] Song X et al (2021) Effect of Endoplasmic reticulum stress-induced apoptosis in the role of periodontitis on vascular calcification in a rat model. J Mol Histol 52(5):1097–1104 34480678 10.1007/s10735-021-10015-z

[123] Zhu Y et al (2018) Advanced glycation end products accelerate calcification in VSMCs through HIF-1α/PDK4 activation and suppress glucose metabolism. Sci Rep 8(1):13730 30213959 10.1038/s41598-018-31877-6 PMC6137084

[124] Mokas S et al (2016) Hypoxia-inducible factor-1 plays a role in phosphate-induced vascular smooth muscle cell calcification. Kidney Int 90(3):598–609 27470678 10.1016/j.kint.2016.05.020

[125] Balogh E et al (2019) Hypoxia triggers osteochondrogenic differentiation of vascular smooth muscle cells in an HIF-1 (hypoxia-inducible factor 1)–dependent and reactive oxygen species–dependent manner. Arterioscler Thromb Vasc Biol 39(6):1088–1099 31070451 10.1161/ATVBAHA.119.312509

[126] Csiki DM et al (2023) Hypoxia-inducible factor activation promotes osteogenic transition of valve interstitial cells and accelerates aortic valve calcification in a mice model of chronic kidney disease. Front Cardiovasc Med 10:1168339 37332579 10.3389/fcvm.2023.1168339 PMC10272757

[127] Tóth A et al (2022) Daprodustat accelerates high phosphate-induced calcification through the activation of HIF-1 signaling. Front Pharmacol 13:798053 35222025 10.3389/fphar.2022.798053 PMC8867606

[128] Adelnia H et al (2023) Metal ion chelation of Poly (aspartic acid): from scale Inhibition to therapeutic potentials. Int J Biol Macromol 229:974–993 36584782 10.1016/j.ijbiomac.2022.12.256

[129] Adelnia H et al (2021) Poly (aspartic acid) in biomedical applications: from polymerization, modification, properties, degradation, and biocompatibility to applications. ACS Biomaterials Sci Eng 7(6):2083–2105 10.1021/acsbiomaterials.1c00150 33797239

[130] Jaminon AM et al (2020) Matrix Gla protein is an independent predictor of both intimal and medial vascular calcification in chronic kidney disease. Sci Rep 10(1):1–9 32313061 10.1038/s41598-020-63013-8 PMC7171129

[131] Barrett H et al (2018) Is matrix Gla protein associated with vascular calcification? A systematic review. Nutrients 10(4):415 29584693 10.3390/nu10040415 PMC5946200

[132] Roumeliotis S et al (2020) Vascular calcification in chronic kidney disease: the role of vitamin K-dependent matrix Gla protein. Front Med 7:154 10.3389/fmed.2020.00154 PMC7193028 32391368

[133] Bjørklund G et al (2020) The role of matrix Gla protein (MGP) in vascular calcification. Curr Med Chem 27(10):1647–1660 30009696 10.2174/0929867325666180716104159

[134] Puzantian H et al (2018) Circulating dephospho-uncarboxylated matrix Gla-protein is associated with kidney dysfunction and arterial stiffness. Am J Hypertens 31(9):988–994 29788226 10.1093/ajh/hpy079 PMC6077812

[135] Jespersen T et al (2020) Uncarboxylated matrix Gla-protein: a biomarker of vitamin K status and cardiovascular risk. Clin Biochem 83:49–56 32422228 10.1016/j.clinbiochem.2020.05.005

[136] Zebboudj AF, Imura M, Boström K (2002) Matrix GLA protein, a regulatory protein for bone morphogenetic protein-2. J Biol Chem 277(6):4388–4394 11741887 10.1074/jbc.M109683200

[137] Schurgers LJ, Uitto J, Reutelingsperger CP (2013) Vitamin K-dependent carboxylation of matrix Gla-protein: a crucial switch to control ectopic mineralization. Trends Mol Med 19(4):217–226 23375872 10.1016/j.molmed.2012.12.008

[138] Kapustin AN et al (2011) Calcium regulates key components of vascular smooth muscle cell–derived matrix vesicles to enhance mineralization. Circul Res 109(1):e1–e12 10.1161/CIRCRESAHA.110.238808 21566214

[139] Ashizawa N et al (1996) Osteopontin is produced by rat cardiac fibroblasts and mediates A (II)-induced DNA synthesis and collagen gel contraction. J Clin Investig 98(10):2218–2227 8941637 10.1172/JCI119031 PMC507670

[140] Ikeda T et al (1993) Osteopontin mRNA is expressed by smooth muscle-derived foam cells in human atherosclerotic lesions of the aorta. J Clin Investig 92(6):2814–2820 8254036 10.1172/JCI116901 PMC288482

[141] Murry CE et al (1994) Macrophages express osteopontin during repair of myocardial necrosis. Am J Pathol 145(6):1450 7992848 PMC1887495

[142] Giachelli CM, Steitz S (2000) Osteopontin: a versatile regulator of inflammation and biomineralization. Matrix Biol 19(7):615–622 11102750 10.1016/s0945-053x(00)00108-6

[143] Wang L et al (2006) Modulation of calcium oxalate crystallization by linear aspartic acid-rich peptides. Langmuir 22(17):7279–7285 16893227 10.1021/la060897z

[144] Steitz SA et al (2002) Osteopontin inhibits mineral deposition and promotes regression of ectopic calcification. Am J Pathol 161(6):2035–2046 12466120 10.1016/S0002-9440(10)64482-3 PMC1850905

[145] Paloian NJ, Leaf EM, Giachelli CM (2016) Osteopontin protects against high phosphate-induced nephrocalcinosis and vascular calcification. Kidney Int 89(5):1027–1036 27083280 10.1016/j.kint.2015.12.046 PMC4834144

[146] Giachelli CM et al (2005) Regulation of vascular calcification: roles of phosphate and osteopontin. Circul Res 96(7):717–722 10.1161/01.RES.0000161997.24797.c0 15831823

[147] Speer MY et al (2002) Inactivation of the osteopontin gene enhances vascular calcification of matrix Gla protein–deficient mice: evidence for osteopontin as an inducible inhibitor of vascular calcification in vivo. J Exp Med 196(8):1047–1055 12391016 10.1084/jem.20020911 PMC2194039

[148] Boskey A et al (2002) Osteopontin deficiency increases mineral content and mineral crystallinity in mouse bone. Calcif Tissue Int 71(2) 10.1007/s00223-001-1121-z 12073157

[149] Wei R, Wong JPC, Kwok HF (2017) Osteopontin–a promising biomarker for cancer therapy. J Cancer 8(12):2173 28819419 10.7150/jca.20480 PMC5560134

[150] Lamort A-S et al (2019) Osteopontin as a link between inflammation and cancer: the thorax in the spotlight. Cells 8(8):815 31382483 10.3390/cells8080815 PMC6721491

[151] Barchetta I et al (2019) Impaired bone matrix glycoprotein pattern is associated with increased cardio-metabolic risk profile in patients with type 2 diabetes mellitus. J Endocrinol Investig 42(5):513–520 30132286 10.1007/s40618-018-0941-x

[152] Gordin D et al (2014) Osteopontin is a strong predictor of incipient diabetic nephropathy, cardiovascular disease, and all-cause mortality in patients with type 1 diabetes. Diabetes Care 37(9):2593–2600 24969575 10.2337/dc14-0065

[153] Lok ZSY, Lyle AN (2019) Osteopontin in vascular disease: friend or foe? Thromb Vascular Biology 39(4):613–622Arteriosclerosis 10.1161/ATVBAHA.118.311577 PMC6436981 30727754

[154] Christensen B et al (2007) Cell type-specific post-translational modifications of mouse osteopontin are associated with different adhesive properties. J Biol Chem 282(27):19463–19472 17500062 10.1074/jbc.M703055200

[155] Jayaprakash NG, Surolia A (2017) Role of glycosylation in nucleating protein folding and stability. Biochem J 474(14):2333–2347 28673927 10.1042/BCJ20170111

[156] Christensen B et al (2016) Transglutaminase 2-catalyzed intramolecular cross-linking of osteopontin. Biochemistry 55(2):294–303 26678563 10.1021/acs.biochem.5b01153

[157] Dalas E et al (2008) The effect of leucine on the crystal growth of calcium phosphate. J Mater Science: Mater Med 19(1):277–282 10.1007/s10856-006-0050-9 17597364

[158] Guo S, Ward MD, Wesson JA (2002) Direct visualization of calcium oxalate monohydrate crystallization and dissolution with atomic force microscopy and the role of polymeric additives. Langmuir 18(11):4284–4291

[159] Rimer JD et al (2017) The role of macromolecules in the formation of kidney stones. Urolithiasis 45(1):57–74 27913854 10.1007/s00240-016-0948-8 PMC5253101

[160] Sheng X, Ward MD, Wesson JA (2003) Adhesion between molecules and calcium oxalate crystals: critical interactions in kidney stone formation. J Am Chem Soc 125(10):2854–2855 12617634 10.1021/ja029575h

[161] El-Abbadi M, Giachelli CM (2007) Mechanisms of vascular calcification. Adv Chronic Kidney Dis 14(1):54–66 17200044 10.1053/j.ackd.2006.10.007

[162] Pal D et al (2012) Fetuin-A acts as an endogenous ligand of TLR4 to promote lipid-induced insulin resistance. Nat Med 18(8):1279 22842477 10.1038/nm.2851

[163] Schinke T et al (1996) The serum protein α2-HS glycoprotein/fetuin inhibits apatite formation in vitro and in mineralizing calvaria cells a possible role in mineralization and calcium homeostasis. J Biol Chem 271(34):20789–20796 8702833 10.1074/jbc.271.34.20789

[164] Heiss A et al (2003) Structural basis of calcification Inhibition by α2-HS glycoprotein/fetuin-A: formation of colloidal calciprotein particles. J Biol Chem 278(15):13333–13341 12556469 10.1074/jbc.M210868200

[165] Reynolds JL et al (2005) Multifunctional roles for serum protein fetuin-a in Inhibition of human vascular smooth muscle cell calcification. J Am Soc Nephrol 16(10):2920–2930 16093453 10.1681/ASN.2004100895

[166] Schäfer C et al (2003) The serum protein α 2–Heremans-Schmid glycoprotein/fetuin-A is a systemically acting inhibitor of ectopic calcification. J Clin Investig 112(3):357–366 12897203 10.1172/JCI17202 PMC166290

[167] Price PA et al (2004) Serum levels of the fetuin-mineral complex correlate with artery calcification in the rat. J Biol Chem 279(3):1594–1600 14578360 10.1074/jbc.M305199200

[168] Ketteler M et al (2003) Association of low fetuin-A (AHSG) concentrations in serum with cardiovascular mortality in patients on dialysis: a cross-sectional study. Lancet 361(9360):827–833 12642050 10.1016/S0140-6736(03)12710-9

[169] Villa-Bellosta R, O’Neill WC (2018) Pyrophosphate deficiency in vascular calcification. Kidney Int 93(6):1293–1297 29580636 10.1016/j.kint.2017.11.035

[170] Villa-Bellosta R (2018) Synthesis of extracellular pyrophosphate increases in vascular smooth muscle cells during phosphate-induced calcification. Arterioscler Thromb Vasc Biol 38(9):2137–2147 30002059 10.1161/ATVBAHA.118.311444

[171] Lomashvili KA et al (2014) Vascular calcification is dependent on plasma levels of pyrophosphate. Kidney Int 85(6):1351–1356 24717293 10.1038/ki.2013.521 PMC4308968

[172] O’neill WC et al (2011) Treatment with pyrophosphate inhibits uremic vascular calcification. Kidney Int 79(5):512–517 21124302 10.1038/ki.2010.461 PMC3183997

[173] Kozák E et al (2021) Oral supplementation of inorganic pyrophosphate in Pseudoxanthoma elasticum. Exp Dermatol 10.1111/exd.14498 PMC8977233 34758173

[174] Lomashvili KA et al (2004) Phosphate-induced vascular calcification: role of pyrophosphate and osteopontin. J Am Soc Nephrol 15(6):1392–1401 15153550 10.1097/01.asn.0000128955.83129.9c

[175] Glatz AC et al (2006) Idiopathic infantile arterial calcification: two case reports, a review of the literature and a role for cardiac transplantation. Pediatr Transplant 10(2):225–233 16573612 10.1111/j.1399-3046.2005.00414.x

[176] Kereiakes DJ et al (2021) Principles of intravascular lithotripsy for calcific plaque modification. Cardiovasc Interventions 14(12):1275–1292 10.1016/j.jcin.2021.03.036 34167671

[177] Hutchison AJ, Smith CP, Brenchley PE (2011) Pharmacology, efficacy and safety of oral phosphate binders. Nat Rev Nephrol 7(10):578 21894188 10.1038/nrneph.2011.112

[178] Cernaro V et al (2016) Phosphate binders for the treatment of chronic kidney disease: role of iron oxyhydroxide. Int J Nephrol Renovascular Disease 9:11 10.2147/IJNRD.S78040 PMC4749089 26893577

[179] Katsumata K et al (2003) Sevelamer hydrochloride prevents ectopic calcification and renal osteodystrophy in chronic renal failure rats. Kidney Int 64(2):441–450 12846739 10.1046/j.1523-1755.2003.00126.x

[180] Sekercioglu N et al (2016) Comparative effectiveness of phosphate binders in patients with chronic kidney disease: a systematic review and network meta-analysis. PLoS ONE 11(6):e0156891 27276077 10.1371/journal.pone.0156891 PMC4898688

[181] Jamal SA et al (2013) Effect of calcium-based versus non-calcium-based phosphate binders on mortality in patients with chronic kidney disease: an updated systematic review and meta-analysis. Lancet 382(9900):1268–1277 23870817 10.1016/S0140-6736(13)60897-1

[182] Qunibi W et al (2008) A 1-year randomized trial of calcium acetate versus Sevelamer on progression of coronary artery calcification in Hemodialysis patients with comparable lipid control: the calcium acetate renagel Evaluation-2 (CARE-2) study. Am J Kidney Dis 51(6):952–965 18423809 10.1053/j.ajkd.2008.02.298

[183] Chertow GM et al (2002) Sevelamer attenuates the progression of coronary and aortic calcification in Hemodialysis patients. Kidney Int 62(1):245–252 12081584 10.1046/j.1523-1755.2002.00434.x

[184] Wang C et al (2015) New conclusions regarding comparison of Sevelamer and calcium-based phosphate binders in coronary-artery calcification for Dialysis patients: a meta-analysis of randomized controlled trials. PLoS ONE 10(7):e0133938 26230677 10.1371/journal.pone.0133938 PMC4521824

[185] Block G et al (2007) Mortality effect of coronary calcification and phosphate binder choice in incident Hemodialysis patients. Kidney Int 71(5):438–441 17200680 10.1038/sj.ki.5002059

[186] Mason DL et al (2022) Effects of Sevelamer carbonate versus calcium acetate on vascular calcification, inflammation, and endothelial dysfunction in chronic kidney disease. Clin Transl Sci 15(2):353–360 34599865 10.1111/cts.13151 PMC8841464

[187] Peter WLS, Wazny LD, Weinhandl ED (2018) Phosphate-binder use in US Dialysis patients: Prevalence, costs, evidence, and policies. Am J Kidney Dis 71(2):246–253 29195858 10.1053/j.ajkd.2017.09.007

[188] Massy ZA, Drüeke TB (2012) Magnesium and outcomes in patients with chronic kidney disease: focus on vascular calcification, atherosclerosis and survival. Clin Kidney J 5(Suppl1):i52–i61 26069821 10.1093/ndtplus/sfr167 PMC4455827

[189] Farzadi A et al (2014) Magnesium incorporated hydroxyapatite: synthesis and structural properties characterization. Ceram Int 40(4):6021–6029

[190] Kanzaki N et al (2000) Inhibitory effect of magnesium and zinc on crystallization kinetics of hydroxyapatite (0001) face. J Phys Chem B 104(17):4189–4194

[191] ter Braake AD et al (2018) Magnesium prevents vascular calcification in vitro by inhibition of hydroxyapatite crystal formation. Sci Rep 8(1):2069 29391410 10.1038/s41598-018-20241-3 PMC5794996

[192] He Y et al (2005) Transient receptor potential melastatin 7 ion channels regulate magnesium homeostasis in vascular smooth muscle cells: role of angiotensin II. Circul Res 96(2):207–215 10.1161/01.RES.0000152967.88472.3e 15591230

[193] Bressendorff I et al (2017) The effect of magnesium supplementation on vascular calcification in chronic kidney disease—A randomised clinical trial (MAGiCAL-CKD): essential study design and rationale. BMJ Open 7(6):e016795 28645983 10.1136/bmjopen-2017-016795 PMC5726116

[194] Bressendorff I et al (2018) The effect of increasing dialysate magnesium on serum calcification propensity in subjects with end stage kidney disease: a randomized, controlled clinical trial. Clin J Am Soc Nephrol 13(9):1373–1380 30131425 10.2215/CJN.13921217 PMC6140556

[195] Houtman E et al (2021) Characterization of dynamic changes in matrix Gla protein (MGP) gene expression as function of genetic risk alleles, osteoarthritis relevant stimuli, and the vitamin K inhibitor warfarin. Osteoarthr Cartil 10.1016/j.joca.2021.05.001 33984465

[196] Rattazzi M et al (2018) Warfarin, but not rivaroxaban, promotes the calcification of the aortic valve in ApoE–/– mice. Cardiovasc Ther 36(4):e12438 29847020 10.1111/1755-5922.12438

[197] Schurgers LJ et al (2007) Regression of warfarin-induced medial elastocalcinosis by high intake of vitamin K in rats. Blood 109(7):2823–2831 17138823 10.1182/blood-2006-07-035345

[198] Chao C-T et al (2019) Natural and non-natural antioxidative compounds: potential candidates for treatment of vascular calcification. Cell Death Discovery 5(1):1–11 10.1038/s41420-019-0225-z PMC6853969 31754473

[199] Mansouri K et al (2020) Clinical effects of Curcumin in enhancing cancer therapy: A systematic review. BMC Cancer 20(1):1–11 10.1186/s12885-020-07256-8 PMC7446227 32838749

[200] Salehi B et al (2019) The therapeutic potential of curcumin: A review of clinical trials. Eur J Med Chem 163:527–545 30553144 10.1016/j.ejmech.2018.12.016

[201] Valizadeh H et al (2020) Nano-curcumin therapy, a promising method in modulating inflammatory cytokines in COVID-19 patients. Int Immunopharmacol 89:107088 33129099 10.1016/j.intimp.2020.107088 PMC7574843

[202] Liu J et al (2020) Curcumin-crosslinked acellular bovine pericardium for the application of calcification Inhibition heart valves. Biomed Mater 15(4):045002 31972553 10.1088/1748-605X/ab6f46

[203] Stefanska J, Pawliczak R (2008) Apocynin: molecular aptitudes. Mediators Inflamm 2008 10.1155/2008/106507 PMC2593395 19096513

[204] Sethi G et al (2018) Pro-apoptotic and anti-cancer properties of diosgenin: a comprehensive and critical review. Nutrients 10(5):645 29783752 10.3390/nu10050645 PMC5986524

[205] Mody N et al (2001) Oxidative stress modulates osteoblastic differentiation of vascular and bone cells. Free Radic Biol Med 31(4):509–519 11498284 10.1016/s0891-5849(01)00610-4

[206] Peralta-Ramírez A et al (2014) Vitamin E protection of obesity-enhanced vascular calcification in uremic rats. Am J Physiology-Renal Physiol 306(4):F422–F429 10.1152/ajprenal.00355.2013 24370590

[207] Baur JA, Sinclair DA (2006) Therapeutic potential of resveratrol: the in vivo evidence. Nat Rev Drug Discovery 5(6):493–506 16732220 10.1038/nrd2060

[208] Xu D et al (2019) Antioxidant activities of Quercetin and its complexes for medicinal application. Molecules 24(6):1123 30901869 10.3390/molecules24061123 PMC6470739

[209] Patel RV et al (2018) Therapeutic potential of Quercetin as a cardiovascular agent. Eur J Med Chem 155:889–904 29966915 10.1016/j.ejmech.2018.06.053

[210] Lu T-S et al (2012) Induction of intracellular heat-shock protein 72 prevents the development of vascular smooth muscle cell calcification. Cardiovascular Res 96(3):524–532 10.1093/cvr/cvs278 22933322

[211] Beazley KE et al (2013) Transglutaminase inhibitors attenuate vascular calcification in a preclinical model. Arterioscler Thromb Vasc Biol 33(1):43–51 23117658 10.1161/ATVBAHA.112.300260 PMC3544469

[212] Liang Q et al (2018) Quercetin attenuates Ox-LDL-induced calcification in vascular smooth muscle cells by regulating ROS-TLR4 signaling pathway. J South Med Univ 38(8):980–985 10.3969/j.issn.1673-4254.2018.08.13 PMC6744032 30187880

[213] Sun Q et al (2013) Protective effect of ginsenoside Rb1 against intestinal ischemia-reperfusion induced acute renal injury in mice. PLoS ONE 8(12):e80859 24324637 10.1371/journal.pone.0080859 PMC3851764

[214] Michiels CF et al (2016) Spermidine reduces lipid accumulation and necrotic core formation in atherosclerotic plaques via induction of autophagy. Atherosclerosis 251:319–327 27450786 10.1016/j.atherosclerosis.2016.07.899

[215] Chang J et al (2019) Dendrobium candidum protects against diabetic kidney lesions through regulating vascular endothelial growth factor, glucose transporter 1, and connective tissue growth factor expression in rats. J Cell Biochem 120(8):13924–13931 31021475 10.1002/jcb.28666

[216] Yonova DH et al (2014) First impressions of cardiovascular calcification treatment in Hemodialysis patients with a new Dialysis fluid containing sodium thiosulphate (STS). Int J Artif Organs 37(4):308–314 24811185 10.5301/ijao.5000309

[217] Ghiandai G et al (2015) Is the sodium thiosulfate therapy useful for vascular calcification in Dialysis pts? vol 32. Organo Ufficiale Della Societa Italiana di Nefrologia, Giornale Italiano di Nefrologia, 3

[218] Yu Y et al (2016) Effect of sodium thiosulfate on coronary artery calcification in maintenance Hemodialysis patients. Zhonghua Yi Xue Za Zhi 96(46):3724–3728 27998429 10.3760/cma.j.issn.0376-2491.2016.46.007

[219] Mathews SJ et al (2011) Effects of sodium thiosulfate on vascular calcification in end-stage renal disease: a pilot study of feasibility, safety and efficacy. Am J Nephrol 33(2):131–138 21242673 10.1159/000323550 PMC3064860

[220] Darres A et al (2019) The effectiveness of topical cerium nitrate-silver sulfadiazine application on overall outcome in patients with calciphylaxis. Dermatology 235(2):120–129 30605905 10.1159/000493975

[221] Narisawa S et al (2007) Novel inhibitors of alkaline phosphatase suppress vascular smooth muscle cell calcification. J Bone Miner Res 22(11):1700–1710 17638573 10.1359/jbmr.070714

[222] Haarhaus M et al (2017) Alkaline phosphatase: a novel treatment target for cardiovascular disease in CKD. Nat Rev Nephrol 13(7):429–442 28502983 10.1038/nrneph.2017.60

[223] Azpiazu D, Gonzalo S, Villa-Bellosta R (2019) Tissue non-specific alkaline phosphatase and vascular calcification: a potential therapeutic target. Curr Cardiol Rev 15(2):91–95 30381085 10.2174/1573403X14666181031141226 PMC6520574

[224] Goettsch C et al (2016) Sortilin mediates vascular calcification via its recruitment into extracellular vesicles. J Clin Investig 126(4):1323–1336 26950419 10.1172/JCI80851 PMC4811143

[225] Opdebeeck B et al (2021) Chronic kidney Disease-Induced arterial media calcification in rats prevented by tissue Non-Specific alkaline phosphatase substrate supplementation rather than Inhibition of the enzyme. Pharmaceutics 13(8):1138 34452102 10.3390/pharmaceutics13081138 PMC8399849

[226] Salcedo C et al (2019) A phase 1b randomized, placebo-controlled clinical trial with SNF472 in haemodialysis patients. Br J Clin Pharmacol 85(4):796–806 30632182 10.1111/bcp.13863 PMC6422667

[227] Dougall W, Chaisson M (2006) Monoclonal antibody targeting RANKL as a therapy for cancer-induced bone diseases. Clin Calcium 16(4):627–635 16582514

[228] Savinov AY et al (2015) Transgenic overexpression of tissue-nonspecific alkaline phosphatase (TNAP) in vascular endothelium results in generalized arterial calcification. J Am Heart Association 4(12):e002499 10.1161/JAHA.115.002499 PMC4845279 26675253

[229] Hasson D, Shemer H, Sher A (2011) State of the Art of friendly green scale control inhibitors: a review Article. Ind Eng Chem Res 50(12):7601–7607

[230] Arbel A, Katz I, Sarig S (1991) Dissolution of hydroxyapatite by calcium complexing agents. J Cryst Growth 110(4):733–738

[231] Lei Y et al (2013) Efficacy of reversal of aortic calcification by chelating agents. Calcif Tissue Int 93(5):426–435 23963635 10.1007/s00223-013-9780-0 PMC3809012

[232] Ibad A, Khalid R, Thompson PD (2016) Chelation therapy in the treatment of cardiovascular diseases. J Clin Lipidol 10(1):58–62 26892121 10.1016/j.jacl.2015.09.005

[233] Lamas GA et al (2013) Effect of disodium EDTA chelation regimen on cardiovascular events in patients with previous myocardial infarction: the TACT randomized trial. JAMA 309(12):1241–1250 23532240 10.1001/jama.2013.2107 PMC4066975

[234] Maniscalco BS, Taylor KA (2004) Calcification in coronary artery disease can be reversed by EDTA–tetracycline long-term chemotherapy. Pathophysiology 11(2):95–101 15364120 10.1016/j.pathophys.2004.06.001

[235] Anderson TJ et al (2003) Effect of chelation therapy on endothelial function in patients with coronary artery disease: PATCH substudy. J Am Coll Cardiol 41(3):420–425 12575969 10.1016/s0735-1097(02)02770-5

[236] Ng PC et al (2021) Efficacy of oral administration of sodium thiosulfate in a large, swine model of oral cyanide toxicity. J Med Toxicol: 1–8 10.1007/s13181-021-00836-5 PMC8206226 33821433

[237] Cicone JS et al (2004) Successful treatment of calciphylaxis with intravenous sodium thiosulfate. Am J Kidney Dis 43(6):1104–1108 15168392 10.1053/j.ajkd.2004.03.018

[238] Yatzidis H (2004) Absence or decreased endogenous thiosulfaturia: a cause of recurrent calcium nephrolithiasis. Int Urol Nephrol 36(4):587–589 15787343 10.1007/s11255-004-8786-y

[239] Grover P et al (2021) In vivo-wound healing studies of sodium thiosulfate gel in rats. Biomed Pharmacother 140:111797 34098193 10.1016/j.biopha.2021.111797

[240] Adirekkiat S et al (2010) Sodium thiosulfate delays the progression of coronary artery calcification in haemodialysis patients. Nephrol Dialysis Transplantation 25(6):1923–1929 10.1093/ndt/gfp755 20083471

[241] Saengpanit D et al (2018) Effect of sodium thiosulfate on arterial stiffness in end-stage renal disease patients undergoing chronic Hemodialysis (sodium thiosulfate-hemodialysis study): a randomized controlled trial. Nephron 139(3):219–227 29587288 10.1159/000488009

[242] Caffarelli C et al (2017) Bisphosphonates, atherosclerosis and vascular calcification: update and systematic review of clinical studies. Clin Interv Aging 12:1819 29133976 10.2147/CIA.S138002 PMC5669782

[243] Zhang S, Gangal G, Uludağ H (2007) Magic bullets’ for bone diseases: progress in rational design of bone-seeking medicinal agents. Chem Soc Rev 36(3):507–531 17325789 10.1039/b512310k

[244] Francis MD et al (1969) Diphosphonates inhibit formation of calcium phosphate crystals in vitro and pathological calcification in vivo. Science 165(3899):1264–1266 4308521 10.1126/science.165.3899.1264

[245] Nitta K et al (2004) Effects of Cyclic intermittent etidronate therapy on coronary artery calcification in patients receiving long-term Hemodialysis. Am J Kidney Dis 44(4):680–688 15384019

[246] Toussaint ND et al (2010) Effect of alendronate on vascular calcification in CKD stages 3 and 4: a pilot randomized controlled trial. Am J Kidney Dis 56(1):57–68 20347511 10.1053/j.ajkd.2009.12.039

[247] Markowitz GS et al (2001) Collapsing focal segmental glomerulosclerosis following treatment with high-dose pamidronate. J Am Soc Nephrol 12(6):1164–1172 11373339 10.1681/ASN.V1261164

[248] Conte P, Guarneri VJTO (2004) Safety of intravenous and oral bisphosphonates and compliance with dosing regimens. Oncologist 9(Supplement 4):28–37 15459427 10.1634/theoncologist.9-90004-28

[249] Torregrosa J-V et al (2010) Open-label trial: effect of weekly risedronate immediately after transplantation in kidney recipients. Transplantation 89(12):1476–1481 20393402 10.1097/TP.0b013e3181dc13d0

[250] Gonnelli S et al (2014) Effects of intravenous zoledronate and ibandronate on carotid intima-media thickness, lipids and FGF-23 in postmenopausal osteoporotic women. Bone 61:27–32 24389416 10.1016/j.bone.2013.12.017

[251] Montagnani A et al (2003) Changes in serum HDL and LDL cholesterol in patients with paget’s bone disease treated with pamidronate. Bone 32(1):15–19 12584031 10.1016/s8756-3282(02)00924-9

[252] Chen X et al (2023) Preparation of protein-loaded nanoparticles based on Poly (succinimide)-oleylamine for sustained protein release: a two-step nanoprecipitation method. Nanotechnology 35(5):055101 10.1088/1361-6528/ad0592 37863070

[253] Moonshi SS et al (2024) Polysuccinimide-based nanoparticle: A nanocarrier with drug release delay and zero burst release properties for effective theranostics of cancer. Appl Mater Today 37:102150

[254] Chen X et al (2025) Preparation of protein-Encapsulated nanoparticles using Polysuccinimide-Oleylamine for sustained protein release. ACS Appl Nano Mater 8(40):19658–19667

[255] Adelnia H et al (2019) Hydrogels based on Poly (aspartic acid): synthesis and applications. Front Chem 7:755 31799235 10.3389/fchem.2019.00755 PMC6861526

[256] Adelnia H et al (2023) Poly (succinimide) nanoparticles as reservoirs for spontaneous and sustained synthesis of Poly (aspartic acid) under physiological conditions: potential for vascular calcification therapy and oral drug delivery. J Mater Chem B 11(12):2650–2662 36655707 10.1039/d2tb01867e

[257] Chen WR et al (2018) Melatonin attenuates myocardial ischemia/reperfusion injury by inhibiting autophagy via an AMPK/mTOR signaling pathway. Cell Physiol Biochem 47(5):2067–2076 29975938 10.1159/000491474

[258] Fernández A et al (2015) Melatonin and Endoplasmic reticulum stress: relation to autophagy and apoptosis. J Pineal Res 59(3):292–307 26201382 10.1111/jpi.12264

[259] Karageuzyan KG (2005) Oxidative stress in the molecular mechanism of pathogenesis at different diseased States of organism in clinics and experiment. Curr Drug Targets Inflamm Allergy 4:85–98 15720241 10.2174/1568010053622939

[260] Yatzidis H (2004) Absence or decreased endogenous thiosulfaturia: a cause of recurrent calcium nephrolithiasis. Int Urol Nephrol 36:587–589 15787343 10.1007/s11255-004-8786-y

[261] Bian Z et al (2022) The effect of sodium thiosulfate on coronary artery calcification in Hemodialysis patients. ASAIO J 68(3):402–406 34294642 10.1097/MAT.0000000000001531

[262] Djuric P et al (2020) Sodium thiosulphate and progression of vascular calcification in end-stage renal disease patients: a double-blind, randomized, placebo-controlled study. Nephrol Dial Transpl 35(1):162–169 10.1093/ndt/gfz204 31764989

[263] Bressendorff I et al (2023) The effect of magnesium supplementation on vascular calcification in CKD: A randomized clinical trial (MAGiCAL-CKD). J Am Soc Nephrol 34(5):886–894 36749131 10.1681/ASN.0000000000000092 PMC10125639

[264] Oikonomaki T et al (2019) The effect of vitamin K2 supplementation on vascular calcification in haemodialysis patients: a 1-year follow-up randomized trial. Int Urol Nephrol 51(11):2037–2044 31529295 10.1007/s11255-019-02275-2

[265] Pawade TA et al (2021) Effect of denosumab or alendronic acid on the progression of aortic stenosis: a double-blind randomized controlled trial. Circulation 143(25):2418–2427 33913339 10.1161/CIRCULATIONAHA.121.053708 PMC8212878

